# Targeted Inhibition of miRNA Maturation with Morpholinos Reveals a Role for miR-375 in Pancreatic Islet Development

**DOI:** 10.1371/journal.pbio.0050203

**Published:** 2007-07-24

**Authors:** Wigard P Kloosterman, Anne K Lagendijk, René F Ketting, Jon D Moulton, Ronald H. A Plasterk

**Affiliations:** 1 Hubrecht Laboratory-KNAW, Utrecht, The Netherlands; 2 Gene Tools, Philomath, Oregon, United States of America; Oregon State University, United States of America

## Abstract

Several vertebrate microRNAs (miRNAs) have been implicated in cellular processes such as muscle differentiation, synapse function, and insulin secretion. In addition, analysis of Dicer null mutants has shown that miRNAs play a role in tissue morphogenesis. Nonetheless, only a few loss-of-function phenotypes for individual miRNAs have been described to date. Here, we introduce a quick and versatile method to interfere with miRNA function during zebrafish embryonic development. Morpholino oligonucleotides targeting the mature miRNA or the miRNA precursor specifically and temporally knock down miRNAs. Morpholinos can block processing of the primary miRNA (pri-miRNA) or the pre-miRNA, and they can inhibit the activity of the mature miRNA. We used this strategy to knock down 13 miRNAs conserved between zebrafish and mammals. For most miRNAs, this does not result in visible defects, but knockdown of miR-375 causes defects in the morphology of the pancreatic islet. Although the islet is still intact at 24 hours postfertilization, in later stages the islet cells become scattered. This phenotype can be recapitulated by independent control morpholinos targeting other sequences in the miR-375 precursor, excluding off-target effects as cause of the phenotype. The aberrant formation of the endocrine pancreas, caused by miR-375 knockdown, is one of the first loss-of-function phenotypes for an individual miRNA in vertebrate development. The miRNA knockdown strategy presented here will be widely used to unravel miRNA function in zebrafish.

## Author Summary

The striking tissue-specific expression patterns of microRNAs (miRNAs) suggest that they play a role in tissue development. These small RNA molecules (∼22 bases in length) are processed from long primary transcripts (pri-miRNA) and regulate gene expression at the posttranscriptional level. There are hundreds of different miRNAs, many of which are strongly conserved. Vertebrate embryonic development is most easily studied in zebrafish, but genetically disrupting miRNA genes to see which miRNA does what is technically challenging. In this study, we interfere with miRNA function during the first few days of zebrafish embryonic development by introducing specific antisense morpholino oligonucleotides (morpholinos have been used previously to interfere with the synthesis of the much larger mRNAs). We show that morpholinos targeting the miRNA precursor can block processing of the pri-miRNA or directly inhibit the activity of the mature miRNA. We also used morpholinos to study the developmental effects of miRNA knockdown. Although we did not observe gross phenotypic defects for many miRNAs, we found that zebrafish miR-375 is essential for formation of the insulin-secreting pancreatic islet. Loss of miR-375 results in dispersed islet cells by 36 hours postfertilization, representing one of the first vertebrate miRNA loss-of-function phenotypes.

## Abstract 3

The authors show that morpholinos can be used to knock down zebrafish miRNAs, revealing that miR-375 is important for vertebrate pancreas development.

## Introduction

MicroRNAs (miRNAs) have a profound impact on the development of multicellular organisms. Animals lacking the Dicer enzyme, which is responsible for the processing of the precursor miRNA into the mature form, cannot live [1–3]. MiRNA mutants have been described only for Caenorhabditis elegans and *Drosophila,* reviewed in [4]. From these studies, it is clear that invertebrate miRNAs are involved in a variety of cellular processes, such as developmental timing [5,6], apoptosis [7,8], and muscle growth [9]. Analysis of conditional Dicer null alleles in mouse has indicated a general role for miRNAs in morphogenesis of the limb, skin, lung epithelium, and hair follicles [10–13]. Overexpression studies in mouse have implicated specific vertebrate miRNAs in cardiogenesis and limb development [14,15]. In zebrafish, embryos lacking both maternal and zygotic contribution of Dicer have severe brain defects [2]. Strikingly, the brain phenotype of maternal-zygotic Dicer zebrafish can be restored by injection of miR-430, the most abundant miRNA in early zebrafish development. Despite all these studies describing functions for miRNAs in development, no vertebrate miRNA mutant has been described to date. Genetically, it is challenging to obtain mutant miRNA alleles in zebrafish, because their small size makes them less prone to mutations by mutagens, and for many miRNAs, there are multiple alleles in the genome or they reside in families of related sequence.

Temporal inhibition of miRNAs by antisense molecules provides another strategy to study miRNA function. 2′-*O*-methyl oligonucleotides have been successfully used in vitro and in vivo to knock down miRNAs [16–18]. Morpholinos are widely applied to knock down genes in zebrafish development [19] and have recently been used to target mature miR-214 in zebrafish [20]. However, off-target phenotypes are often associated with the use of antisense inhibitors.

Here, we show that morpholinos targeting the miRNA precursor can knock down miRNAs in the zebrafish embryo. Several independent morpholinos can knock down the same miRNA, and these serve as positive controls to filter out off-target effects. Morpholinos can block miRNA maturation at the step of Drosha or Dicer cleavage, and they can inhibit the activity of the mature miRNA. We show that inhibition of miR-375, which is expressed in the pancreatic islet and pituitary gland of the embryo [21], results in dispersed islet cells in later stages of embryonic development, whereas no effects were observed in the pituitary gland. The morpholino-mediated miRNA knockdown strategy presented here, is an extremely fast and well-controlled method to study miRNA function in development.

## Results

### Morpholinos Targeting the Mature miRNA Deplete the Embryo of Specific miRNAs

**Figure 1:**
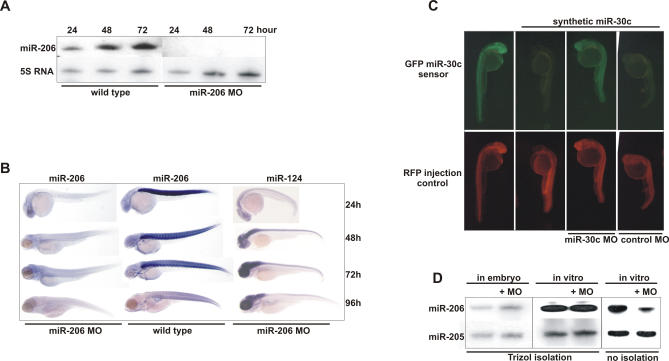
Morpholinos Targeting the Mature miRNA Deplete the Zebrafish Embryo of Specific miRNAs (A) Northern blot for miR-206 in wild-type and MO miR-206–injected embryos at 24, 48, and 72 hpf. 5S RNA serves as a loading control. (B) In situ analysis of miR-206 and miR-124 expression in different stage embryos after injection of MO miR-206. (C) Effect of a morpholino targeting miR-30c on a silencing assay with miR-30c and a responsive GFP sensor construct. (D) In vivo and in vitro effects of a morpholino on the stability and RNA extraction of a synthetic miR-206 duplex. miR-205 serves as a loading control.

Since it is difficult to obtain a genetic mutant for a miRNA in zebrafish, we looked for alternative strategies to deplete the embryo of specific miRNAs. Antisense molecules such as 2′-*O*-methyl and locked nucleic acid (LNA) oligonucleotides have been used to inhibit miRNAs in cell lines [16,18,22], *Drosophila* embryos [23], and adult mice [17]. We tried to use these molecules to inhibit the function of endogenous miRNAs in the zebrafish embryo. Although they can be used to suppress the effects of miRNA overexpression [24], injection of higher concentrations required to obtain good knockdown of endogenous miRNAs resulted in toxic effects, when injecting 1 nl solution at a concentration of approximately 10 μM and 50 μM for LNA and 2′-*O*-methyl oligonucleotides, respectively (unpublished data). Therefore, we switched to morpholinos because these are widely used to inhibit mRNA translation and splicing in zebrafish embryos [19], and have also been shown to target miRNAs in the embryo [2,20,24]. We injected 1 nl of 600 μM morpholino solution with a morpholino complementary to the mature miR-206 in one- or two-cell–stage embryos. Subsequently, embryos were harvested at 24, 48, 72, and 96 hours postfertilization (hpf), and subjected to in situ hybridization and Northern blotting (Figure 1A and 1B). This analysis showed that the mature miRNA signal is suppressed up to 4 d after injection of the morpholino. The knockdown effect was specific for this miRNA; parallel in situ analysis of the same embryos with a probe for miR-124 did not show any effects on expression of this miRNA (Figure 1B). Thus, miRNA detection can be specifically and efficiently suppressed during embryonic and early larval stages of zebrafish development using morpholinos antisense to the mature miRNA.

The zebrafish embryo can be used to monitor the effect of miRNAs on green fluorescent protein (GFP) reporters fused to miRNA target sites [24]. To determine the effect of a morpholino in this assay system, we constructed a GFP reporter for miR-30c and tested it in the presence and absence of a mature miR-30c duplex. Injected miR-30c silences this GFP reporter, which is in line with previous reports using similar strategies in the embryo (Figure 1C) [2,20,24]. Co-injection of the miR-30c duplex and a morpholino targeting mature miR-30c rescues the reporter signal, whereas injection of a control morpholino did not reverse the silencing by miR-30c. These data indicate that a morpholino can block the activity of a mature miRNA duplex in a functional assay .

There are three possible explanations for the observed reduction in the detection signal for a miRNA that is targeted by a morpholino. First, the hybridization of a morpholino could disturb isolation of the miRNA. Second, the morpholino could destabilize the miRNA. Third, the morpholino could inhibit the maturation of the miRNA.

To examine the effect of a morpholino on the isolation of a mature miRNA, we incubated a mature miR-206 duplex and a control duplex (miR-205) with a morpholino against miR-206 in vitro. After isolation, samples were analyzed by Northern blotting for the presence of miR-206 and miR-205. We could still detect miR-206, indicating that there is no effect of the morpholino on the RNA isolation procedure (Figure 1D). However, when morpholino and miRNA duplex were incubated together in vitro and loaded on a denaturing gel without isolation, we observed a decrease in the signal for miR-206, indicating that the morpholino can bind to the miRNA in vitro and still does so in the denaturing gel.

Next, we wanted to know whether a morpholino could affect the stability of a mature miRNA in vivo. Therefore, we injected a mature miR-206 and a control duplex (miR-205) together with a morpholino against miR-206 in the embryo. After incubation for 8 h, RNA was isolated and subjected to Northern blot analysis to probe for injected miR-206 and injected miR-205. In contrast to the data obtained for endogenous miR-206, there was no decrease observed in the amount of injected miR-206 in the morpholino-injected embryos (Figure 1D) (endogenous miR-206 is not yet expressed at this stage).

Since these data show that there is no effect of a morpholino on miRNA isolation or stability, we conclude that morpholinos deplete the embryo of miRNAs by inhibiting miRNA maturation. If this is the case, then we expect morpholinos targeting other regions of the miRNA precursor to act as well as the morpholinos designed against the mature miRNA, and this is indeed what we find (see next section).

### Morpholinos Targeting the miRNA Precursor Interfere with Primary miRNA Processing

Injection of antisense oligos in embryos might result in off-target effects. Thus, phenotypic data retrieved from antisense knockdown experiments should be treated with caution. In *Drosophila,* 2′-*O*-methyl oligo–mediated knockdown of embryonically expressed miRNAs caused defects that clearly differed from the phenotype of the corresponding knockout fly [9,23]. In sea urchin experiments, off-target effects of morpholino knockdowns are well documented, though low incubation temperatures favor off-target interactions [25].

**Figure 2:**
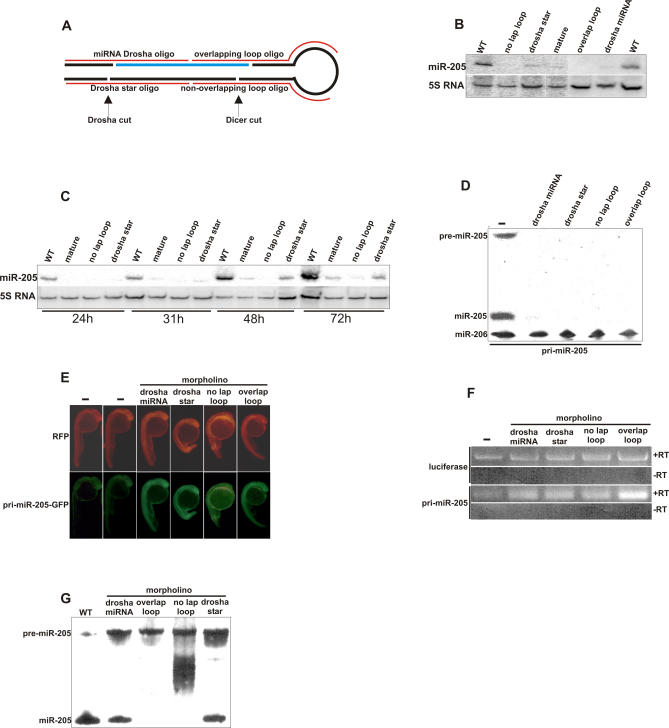
Morpholinos Targeting the Precursor miRNA Interfere with miRNA Maturation (A) Design of morpholinos targeting the precursor miRNA. (B) Northern blot analysis of miR-205 in 30-h-old embryos injected with different morpholinos against pri-miR-205. 5S RNA serves as a loading control. (C) Time series of miR-205 expression after injection of mature, no lap loop, and drosha star morpholinos against pri-miR-205. (D) Northern blot analysis of miR-205 derived from embryos injected with a GFP-pri-miR-205 transcript and four different morpholinos targeting pri-miR-205. Co-injected miR-206 serves as an injection and loading control. Embryos were collected 8 h after injection. (E) GFP expression in 24-h embryos injected with morpholinos and a GFP-pri-miR-205 construct as used in (C). Pri-miR-205 is positioned just upstream of the polyA signal in the 3′ UTR of the GFP mRNA. Red fluorescent protein (RFP) serves as an injection control. (F) RT-PCR analysis of injected GFP-pri-miR-205 mRNA with (+) and without (−) co-injected morpholinos. Luciferase serves a an injection control. Embryos were collected 8 h after injection. (G) Northern analysis of the effect of morpholinos on an injected miR-205 precursor. Embryos were collected 8 h after injection. WT, wild type.

To filter out off-target effects, we sought a control strategy that would allow us to compare effects of morpholinos with independent sequences targeted to the same miRNA. Because our data on morpholinos targeting the mature miRNA suggested that miRNA biogenesis might be affected, we designed morpholinos targeting the Drosha and Dicer cleavage sites of the precursor miRNA (Figure 2A). We decided to test this strategy on miR-205, since it is expressed relatively early, and there are only two, but identical, copies in the fish genome. Four different morpholinos were designed to inhibit miR-205 biogenesis: two targeting the Drosha cleavage site complementary to either the 5′ or 3′ arm of the stem, and two morpholinos similarly targeting the Dicer cleavage site (Figure S1). These morpholinos were injected under similar conditions as described for miR-206 and compared to the morpholino targeting mature miR-205. Interestingly, all five morpholinos induced complete or near-complete loss of miR-205 (Figure 2B).

Many miRNAs are highly expressed during later stages of embryonic development [21]. Therefore, we tested how long the effect of the morpholinos would last. Although for this series of morpholinos the knockdown is best at 24 hpf, the effect is still significant up to 72 hpf (Figure 2C).

Next, we tested a similar series of morpholinos against the miR-30c precursor and analyzed miR-30c expression by Northern blotting (Figure S2). However, we only observed knockdown for the morpholino targeting mature miR-30c, but not for the other four morpholinos targeting the miR-30c precursor. This could be because miR-30c resides in a family of closely related species, with more sequence variability in the regions outside of the mature miRNA. The precursors of the family members might not all be targeted by these morpholinos (Figure S2). Thus, not all miRNAs are equally prone to knockdown by morpholinos that target the miRNA precursor.

To investigate the effect of morpholinos on exogenously introduced pri-miR-205, we injected mRNA derived from a GFP construct with pri-miR-205 in the 3′ UTR. Again, we could not detect mature miR-205 derived from this construct after targeting by morpholinos (Figure 2D). Interestingly, the miR-205 precursor also could not be detected in the embryos co-injected with morpholinos, whereas pre-miR-205 could be detected in the absence of morpholinos (Figure 2D). Because pri-miR-205 was cloned in the 3′ UTR of GFP, we monitored GFP fluorescence after injection of this construct. In the presence of a morpholino, GFP fluorescence increased (Figure 2E), suggesting accumulation of the primary miRNA. Therefore, we performed reverse transcriptase PCR (RT-PCR) on 8-h-old embryos injected with GFP-pri-miR-205 and a control mRNA (luciferase) (Figure 2F). In the presence of a morpholino, the GFP-pri-miR-205 mRNA level is higher compared to control embryos that were not injected with morpholinos. This experiment confirms the GFP data and shows that morpholinos targeting the miRNA precursor inhibit Drosha cleavage.

Next, we tested whether processing of the pre-miRNA might also be inhibited by morpholinos. Therefore, we injected a miR-205 precursor in the one-cell–stage embryo. Northern analysis showed that the precursor was processed into mature miRNA in the embryo (Figure 2G). However, co-injection of the overlap loop and non-overlapping loop morpholinos blocked processing completely. There was only a little effect of morpholinos targeting the Drosha cleavage site, probably because they only partially overlap the precursor.

A similar analysis was performed for miR-375, which is expressed in the pancreatic islet and pituitary gland [21], and has two copies in the zebrafish genome, which differ in the regions outside the mature miRNA.

**Figure 3:**
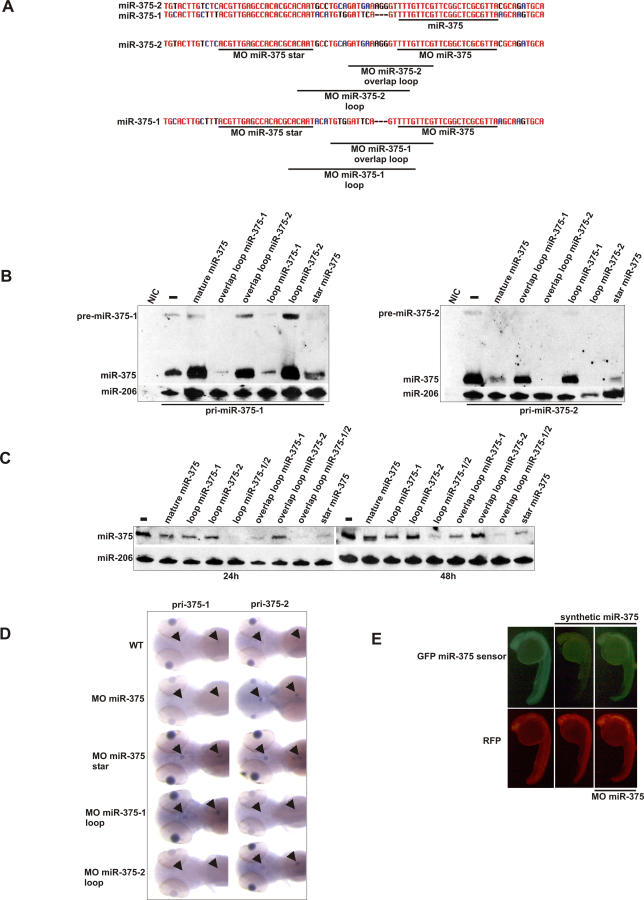
Specific Morpholinos Deplete the Embryo of miR-375 (A) Sequence alignment of the two miR-375 genes from zebrafish and design of morpholinos targeting the dre-miR-375–1 and dre-miR-375–2 precursors. (B) Northern blot analysis of the effect of morpholinos on the expression of miR-375 derived from injected pri-miRNA mRNAs for miR-375–1 and miR-375–2. MO-375–1 overlap loop and loop morpholinos target exclusively the pri-miR-375–1 construct, and MO-375–2 overlap loop and loop morpholinos target exclusively the pri-miR-375–2 construct. Co-injected miR-206 serves as a loading and injection control. Embryos were collected 8 h after injection. (C) Northern blot analysis of the effect of morpholinos on endogenous miR-375 expression at 24 hpf and 48 hpf. MiR-206 serves as loading control. (D) In situ hybridization for pri-miR-375–1 and pri-miR-375–2 on wild-type (WT) and morpholino-injected embryos. Arrowheads indicate the pituitary gland and the pancreatic islet. (E) Analysis of GFP expression in 24-h embryos injected with a miR-375 GFP sensor construct, a synthetic miR-375 duplex and MO miR-375. Red fluorescent protein (RFP) serves as an injection control. NIC, noninjected control.

Overlap loop and loop morpholinos were designed for both miR-375–1 and miR-375–2, and a morpholino against the miRNA star sequence could be used to target both copies of miR-375 simultaneously (Figure 3A). The efficacy of all morpholinos was assessed by determining their effect on injected pri-miR-375–1 or pri-miR-375–2 transcripts (Figure 3B). As expected, each morpholino targeted the transcript to which it was directed. However, the star miR-375 morpholino did not knock down miR-375 completely. In addition, morpholino oligonucleotide (MO) miR-375 did not interfere with processing of miR-375 from pri-miR-375–1, possibly because this primary transcript forms a more stable hairpin. In all cases, the lack of a signal for mature miR-375 coincided with the absence of pre-miR-375, which could be detected in the absence of a complementary morpholino.

Next, all morpholinos were injected separately and in combination, and embryos were subjected to Northern blotting to determine endogenous miR-375 expression at 24 and 48 hpf (Figure 3C). In contrast to the results obtained by in situ hybridization (see last section), the morpholino to mature miR-375 only slightly decreased the expression of miR-375. However, MO miR-375 could inhibit the activity of a mature miR-375 duplex in a GFP-miR-375-target reporter assay (Figure 3E). The morpholinos targeting only one copy of miR-375 reduced miR-375 expression, with the strongest effect for the morpholinos targeting pri-miR-375–1. However, simultaneous injection of morpholinos targeting pri-miR-375–1 and pri-miR-375–2 completely knocked down mature miR-375, indicating that both transcripts are expressed.

To further determine the contribution of each transcript to mature miR-375 accumulation, we performed in situ hybridization for pri-miR-375–1 and pri-miR-375–2 (Figure 3D). Both transcripts could not be detected in wild-type embryos. However, pri-miR-375–1 was detected in the pancreatic islet and the pituitary gland in embryos injected with the miR-375–1 loop morpholino and the morpholino to miR-375 star. Similarly, pri-miR-375–2 was only detected in embryos injected with the miR-375–2 loop morpholino, the morpholino to miR-375 star and mature miR-375. Thus, both transcripts are expressed in the pituitary gland and the pancreatic islet, similar to miR-1 in the developing mouse heart [15]. Together, this indicates that these morpholinos inhibit primary miRNA processing and result in primary miRNA accumulation, as we described for miR-205.

In conclusion, our data demonstrate that morpholinos targeting the miRNA precursor can interfere with primary miRNA processing at either the Drosha or Dicer cleavage step and that morpholinos targeting the mature miRNA can inhibit their activity in a functional assay. Taken together, our data show that different morpholinos targeting the same miRNA may serve as positive controls for miRNA knockdown phenotypes in the embryo.

### Knockdown of Many miRNAs Does Not Result in Any Observed Developmental Defects

**Figure 4:**
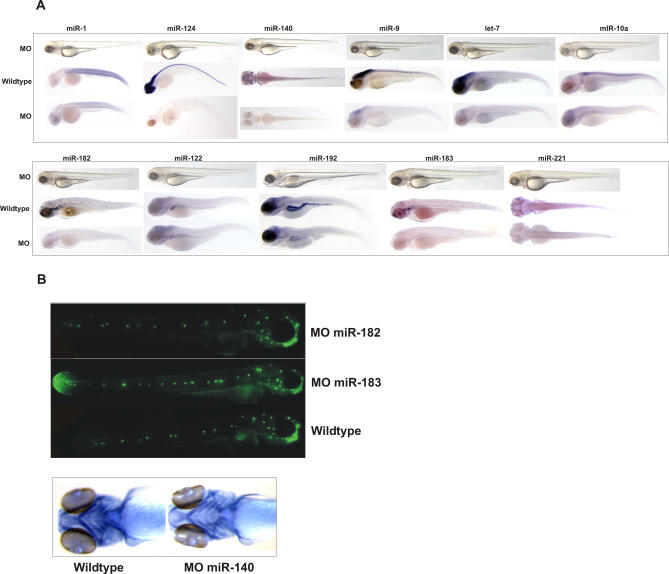
Knockdown of Many miRNAs Does Not Affect Zebrafish Embryonic Development (A) Phenotypes and in situ analysis of 3- and 4-d-old embryos after injection of morpholinos against 11 different mature miRNAs. (B) Daspei staining of 72-h-old embryos injected with MO miR-182 and MO miR-183, and wild-type control (upper panel). Alcian Blue staining of 72-h-old embryos injected with MO miR-140 and noninjected control (lower panel).

To identify functions for individual miRNAs in zebrafish embryonic development, we knocked down a series of 11 conserved vertebrate miRNAs (Table S1) and analyzed their expression after morpholino knockdown. Injected embryos were monitored phenotypically by microscopic observation until four days postfertilization (dpf). Knockdown of most miRNAs resulted in loss of in situ staining for the respective miRNA. However, we could not observe gross morphological malformations after knockdown of these miRNAs (Figure 4A). Therefore, we analyzed embryos injected with morpholinos against miR-182, miR-183, or miR-140 in more detail, because we could easily stain the tissues that express these miRNAs (Figure 4B). Embryos injected with morpholinos against miR-182 or miR-183, which are expressed in the lateral line neuromasts and hair cells of the inner ear, were treated with DASPEI, which stains hair cells. Embryos injected with a morpholino against miR-140, which is expressed in cartilage, were subjected to Alcian Blue staining, a cartilage marker. However, staining of these specific cell types that express the miRNA did not uncover any defects upon knockdown (Figure 4B).

In conclusion, knockdown of many miRNAs does not appear to significantly affect zebrafish embryonic development, at least not to the extent that can be visualized by the methods used in these examples.

### Knockdown of miR-375 Affects Pancreatic Islet Morphology

MiR-375 is known to be expressed in the pancreatic islet and the pituitary gland, and was first isolated from pancreatic beta cells [21,26]. This miRNA is conserved in vertebrates and may regulate insulin secretion by inhibiting myotrophin [26].

**Figure 5:**
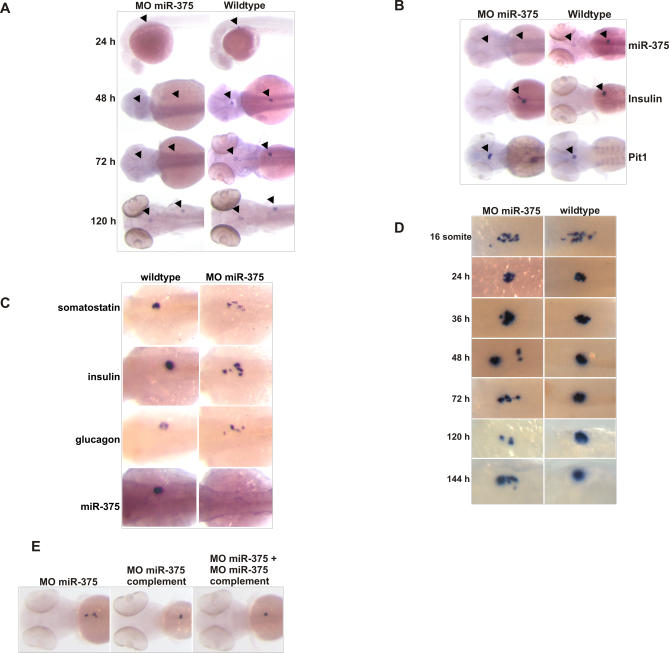
Knockdown of miR-375 Results in Aberrant Migration of Pancreatic Islet Cells (A) In situ analysis of miR-375 knockdown in MO miR-375–injected embryos and noninjected controls at 24, 48, 72, and 120 hpf. Arrowheads indicate the pituitary gland and the pancreatic islet. (B) In situ analysis of the pancreatic islet (insulin staining) and the pituitary gland (pit1 staining) in miR-375 morphants and noninjected controls. Arrowheads indicate the pituitary gland and the pancreatic islet. (C) In situ analysis of pancreatic islet development in wild-type and morphant embryos using insulin, somatostatin, and glucagon as markers. (D) Time series of insulin expression in wild-type and morphant embryos injected with MO miR-375. (E) Insulin expression in 72-hpf embryos injected with MO miR-375 and a complementary morpholino.

We injected a morpholino against mature miR-375 into the one-cell–stage embryo. This morpholino effectively knocked down miR-375 in the first 4 d of development (Figure 5A), and it could also block the activity of an injected miR-375 duplex, as monitored by its effect on a GFP reporter silenced by miR-375 (Figure 3E).

During the first 5 dpf, there was no clear developmental defect except for a general delay in development. At around 7 dpf, approximately 80% of the injected embryos died. Next, we analyzed the development of both the pituitary gland and the pancreatic islet, by in situ hybridization with pit1 and insulin markers. This analysis revealed no change in the formation of the pituitary gland (Figure 5B). However, analysis of insulin expression showed a striking malformation of the islet cells in 3-d-old morphant embryos (Figure 5B). Wild-type embryos have a single islet at the right side of the midline, whereas the miR-375 knockdown embryos have dispersed insulin-positive cells. The effect is sequence specific, because a morpholino complementary to the mature miR-375 morpholino inhibited the pancreatic islet phenotype (Figure 5E).

The pancreatic islet consists of four cell types, α, β, δ, and PP, expressing glucagon, insulin, somatostatin, and pancreatic polypeptide, respectively. Insulin is the first hormone expressed, and somatostatin co-localizes partially with insulin, whereas glucagon-expressing cells are distinct [27]. A more detailed analysis using somatostatin and glucagon as marker genes revealed a similar pattern of scattered islet cells in the miR-375 morphant (Figure 5C).

In zebrafish, insulin is first expressed at the 12-somite stage in a few scattered cells located at the midline, dorsal to the yolk [28]. Insulin-positive cells migrate posteriorly and converge medially to form an islet by 24 hpf. To look at the development of the pancreatic islet in time, we collected MO miR-375 and noninjected control embryos at different stages, and investigated the expression of insulin (Figure 5D). At the 16-somite stage, insulin-positive cells are scattered at the midline in both noninjected and MO miR-375–injected embryos, and a presumptive islet is formed by 24 hpf. Subsequently, when the insulin-positive islet is moving to the right side of the embryo in later stages, the islet breaks apart and insulin-positive cells become scattered in morphant embryos (Figure 5D). Also, in later stages, the phenotype persists, although miR-375 is re-expressed at approximately 5 dpf in morpholino-injected embryos (Figure 5A).

**Figure 6:**
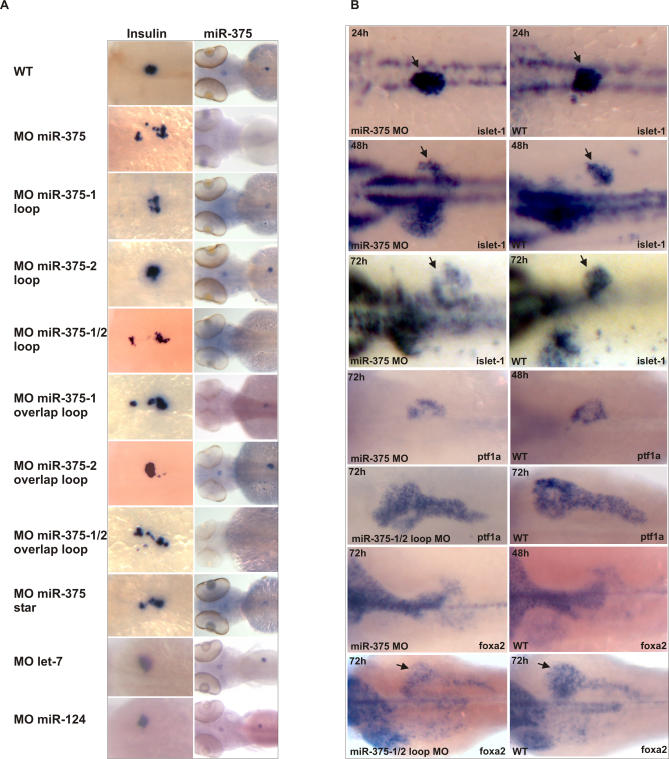
Specific Effects of miR-375 Knockdown on the Development of the Endocrine Pancreas (A) In situ analysis of miR-375 and insulin expression in 72-hpf embryos injected with morpholinos against the miR-375 precursor and negative control morpholinos for let-7 and miR-124. (B) Expression of islet1, foxa2, and ptf1a in wild-type and miR-375 knockdown embryos. Arrows indicate the pancreatic islet. WT, wild type

Next, we analyzed the effect of all miR-375 control morpholinos described in the previous section, by staining for insulin (Figure 6A). Both the dispersion phenotype and the knockdown were striking for embryos injected with MO miR-375. Injection of the overlap loop and loop morpholinos targeting pri-miR-375–1 also resulted in scattered insulin-positive cells at 72 hpf, although the effect was weaker compared to MO miR-375. The miR-375–2 loop and overlap loop morpholinos hardly induced any scattering of insulin-positive cells, whereas the effect was very strong in embryos injected with morpholinos to pri-miR-375–1 and −2 simultaneously. The effect of the miR-375 star morpholino on insulin-positive cells was moderate compared to MO miR-375.

To further prove the specificity of the pancreatic islet phenotype, we injected two control morpholinos against let-7 and miR-124 and analyzed these for miR-375 and insulin expression. None of these control morpholinos showed loss of miR-375 expression or abnormal development of the islet cells (Figure 6A).

Next, we analyzed miR-375 knockdown embryos with markers staining the endocrine or exocrine pancreas (Figure 6B). Similar to insulin staining, islet1 expression showed dispersed islet cells in embryos of 48 hpf and 72 hpf, but not 24 hpf. Embryos injected with MO miR-375 exhibited delayed development of the exocrine pancreas, liver, and gut as shown by ptf1a and foxa2 staining. At 72 hpf, these markers showed a similar pattern in MO miR-375–injected embryos as in noninjected embryos at 48 hpf. However, co-injection of miR-375–1/2 loop morpholinos did not delay development of the exocrine significantly, but these embryos still displayed the scattered insulin-positive cells (Figure 6A). This shows that loss of miR-375 mainly results in malformation of the endocrine pancreas, whereas surrounding tissues that do not express miR-375 are not affected.

## Discussion

Functional data on miRNAs in vertebrate development have been obtained mainly from overexpression studies and analysis of conditional Dicer knockouts. For example, the role of miR-430 in zebrafish brain morphogenesis has become clear from experiments that rescued Dicer null mutants by injection of an miRNA duplex that mimicked a miR-430 family member [2].

MiRNA expression can be conveniently studied in zebrafish embryos. However, dissecting miRNA function by disrupting miRNA genes is difficult in zebrafish, because the miRNA is too small to efficiently search for mutations by a target-selected mutagenesis approach [29]. In addition, it is unclear what such point mutations would do to processing or function of the miRNA.

It has been shown previously that morpholinos can target miRNAs in the zebrafish embryo [20,24]. In a recent study, mature miR-214 was targeted by a morpholino in zebrafish, and this resulted in a change in somite shape, reminiscent of attenuated hedgehog signaling [20]. Although the phenotype could be rescued by simultaneous inhibition of a negative regulator of hedgehog signaling, no positive control morpholinos were reported that could mimic the phenotype. In addition, data were lacking that showed an effect of the morpholino on endogenous miR-214 levels.

The results in this paper show that morpholinos targeting the miRNA precursor form a reliable and efficient tool to deplete the embryo of miRNAs during the first 4 d of development, when most organ systems are formed and miRNAs are expressed. We have shown that miRNA expression can be inhibited by targeting the mature miRNA, the precursor miRNA or the primary miRNA. Our data show that such morpholinos can inhibit miRNA processing at the Drosha cleavage step or the Dicer cleavage step, probably by steric blocking, although the exact mechanism is unclear. In addition, morpholinos targeting the mature miRNA can inhibit their activity, probably by preventing binding to a target mRNA.

We used morpholinos targeting the mature miRNA for a set of 13 conserved vertebrate miRNAs to identify their developmental functions. By microscopic analysis, we could not observe clear defects associated with loss of 11 of these miRNAs during the first 4 d of embryonic development, although in situ hybridization revealed specific loss of most knocked-down miRNAs. Because all the targeted miRNAs are expressed in very specific tissues and we did not investigate most morphants in much detail by marker analysis, we may have missed subtle defects. In addition, many miRNAs reside in families of related sequence (e.g., let-7 and miR-182), and these should possibly be targeted simultaneously by different morpholinos to obtain a biological effect. Furthermore, in those instances in which miRNAs of unrelated sequence target a similar set of mRNAs when expressed in the same tissue [21], removing only one miRNA might not have a profound impact on transcript levels or expression. Finally, microarray analysis and computational predictions have shown that a single miRNA may regulate hundreds of mRNAs [30,31], but that some miRNAs act as a backup for mRNAs that are already repressed transcriptionally [32]. Thus, knockdown of such miRNAs might not dramatically affect gene expression, but ensure robustness of protein interaction networks as for example miR-7 in *Drosophila* [33].

In zebrafish, there are two copies of miR-375, and in human and mouse only one copy has been identified [34]. To verify the miR-375 knockdown phenotype, we designed control morpholinos targeting both precursors simultaneously (MO miR-375 star) and separately. Complete knockdown was only observed in those instances in which both miR-375 copies were targeted simultaneously. This also led to scattered islet cells, proving the specificity of the phenotype. However, knockdown with miR-375–1/2 loop morpholinos did not delay development as seen in the knockdown with the mature miR-375 morpholino. This shows the strength of using control morpholinos and excludes the delayed development as a relevant miR-375 loss-of-function phenotype. A moderate version of the phenotype was also observed in embryos injected with a morpholino specifically targeting miR-375–1. Thus, a reduction in the level of miR-375 already disturbs islet integrity. Similar to mouse miR-1 [15], miR-375 copies survived evolution and are expressed similarly in time and space, probably to ensure the high intracellular concentration of miR-375 necessary to repress many weakly binding targets.

In a forward genetic screen, several mutants were identified with improper development of the endocrine pancreas [35]. These mutants fall into three classes: (1) mutants with severely reduced insulin expression; (2) mutants with reduced insulin expression and abnormal islet morphology; and (3) mutants with normal levels of insulin expression and abnormal islet morphology. However, in all of these mutants, islet cells do not merge into an islet from their first appearance at approximately the 14-somite stage. Our miR-375 knockdown phenotype differs from this, because in the first instance, an islet is formed at approximately 24 hpf, but in later stages, the islet falls apart into small groups of cells. This rules out a general role for miR-375 in early endocrine formation as is seen for Wnt5 [36], but rather indicates a role in maintenance of tissue identity, which is assumed to be a general function of miRNAs in development [21]. It is as yet unclear which miR-375 targets are involved in the phenotype. Work in cell lines has implicated miR-375 in insulin secretion by targeting *myotrophin* [26]. The zebrafish homolog of *myotrophin* also contains a seven-nucleotide seed match to miR-375 (unpublished data), but future studies should reveal whether this target or many other predicted targets are relevant to the phenotype. The specific expression of miR-375 in the pancreatic islet and its implication in insulin secretion make it a candidate drug target in diabetes, e.g., to influence insulin levels in the blood. However, our data show that if miR-375 is used as a drug target, developmental side effects need to be taken into account.

## Materials and Methods

### Morpholino and miRNA injections.

Morpholinos were obtained from Gene Tools LLC (http://www.gene-tools.com) and dissolved to a concentration of 5 mM in water. Morpholinos were injected into one- or two-cell–stage embryos at concentrations between 200 μM and 1,000 μM, and per embryo, one nl of morpholino solution was injected.

RNA oligos (Table S2) were obtained from Sigma (http://www.sigmaaldrich.com) and dissolved to a concentration of 100 μM in distilled water. Oligos were annealed using a 5x buffer containing 30 mM HEPES-KOH (pH 7.4), 100 mM KCl, 2 mM MgCl_2_, and 50 mM NH_4_Ac. Typically, 1 nl of a 10 μM miRNA duplex solution was injected.

All morpholino sequences used in this study are listed in Table S1.

### Construction of miR-30c and miR-375 GFP reporters and pri-miRNA constructs.

The miR-30c and miR-375 reporter constructs were made by cloning two annealed oligos containing two perfectly complementary miRNA target sites into pCS2 (Clontech, http://www.clontech.com) containing a *gfp* gene between BamHI and ClaI restriction sites. A construct containing pri-miR-205 was made by amplifying a genomic region (801 base pairs) containing the miR-205 precursor (miR-205-hairpinF ggcattgaattcataaCCTCTTACCTGCATGACCTG; miR-205-hairpinR ggcatttctagaGTGTGTGCGTGTATTCAACC). The resulting PCR fragment was cloned between XbaI and EcoRI restriction sites of PCS2GFP. Pri-miR-375–1 and pri-miR-375–2 constructs were made by amplifying genomic regions containing miR-375–1 and miR-375–2 precursors (WKmiR-375–1F-pCS2 gcccgggatccTGTGTCTTGCAGGAAAAGAG; WKmiR-375–1R-pCS2 attacgaattcTCAAACTCTCCACTGACTGC; and WKmiR-375–2F-pCS2 gcccgggatccGCCCTCCCATTTGACTC; WKmiR-375–2R-pCS2 attacgaattcAATGAGTGCACAAAATGTCC), and cloning of the resulting PCR fragments into the BamHI and EcoRI sites of pCS2. mRNA was synthesized using SP6 RNA polymerase. Luciferase mRNA was derived from pCS2 containing luciferase between BamHI and EcoRI sites.

### In situ hybridization, Northern blotting, and RT-PCR.

In situ hybridization was performed as described previously [37]. LNA probes for miRNA detection were obtained from Exiqon (http://www.exiqon.com) and labeled using terminal transferase and DIG-11-ddUTP. cDNA clones for pri-miR-375–1, pri-miR-375–2, pit1, insulin, somatostatin, and glucagon were used for antisense DIG-labeled probe synthesis by T7 or Sp6 RNA polymerase.

For Northern blotting, total RNA was isolated from ten embryos per sample using Trizol reagent (Invitrogen, http://www.invitrogen.com). RNA was separated on a 15% denaturing polyacrylamide gel. Radiolabeled DNA probes complementary to miRNAs or 5S RNA (atcggacgagatcgggcgta) were used for hybridization at 37 °C. Stringency washes were done twice for 15 min at 37 °C using 2 × SSC 0.2% SDS. Alternatively, DIG-labeled LNA probes were used for hybridization at 60 °C and stringency washes were performed at 50 °C with 2x SSC 0.1% SDS for 30 min and 0.5x SSC 0.1% SDS for 30 min.

For RT-PCR, RNA was isolated with Trizol, treated with DNAse (Promega, http://www.promega.com) and subsequently purified again using Trizol. cDNA was made with a poly dT primer. Primers used for amplification were miR-205-hairpinF and miR-205-hairpinR, and lucF (ATGGAAGACGCCAAAAACATAAAG) and lucR (ATTACATCGATTTACACGGCGATCTTTCC).

### Alcian Blue and Daspei staining.

For Alcian Blue staining, embryos were fixed for 1 h at room temperature in 4% PFA in PBS, rinsed for 5 min in 50% MeOH, and stored overnight in 70% MeOH at 4 °C. Next, embryos were incubated for 5 min in 50% MeOH and for 5 min in 100% EtOH. Embryos were stained at room temperature with Alcian Blue (Sigma) for 90 min with continuous shaking. Subsequently, embryos were rinsed in 80%, 50%, and 25% EtOH for 2 min each and two times in water containing 0.2% Triton and neutralized in 100% Borax solution. Finally, embryos were incubated for 60 min in digest solution (60% Borax solution, 1 mg/ml colleganase-free and elastase-free trypsin, 0.2% trypsin) and stored in 70% glycerol.

Staining of the hair cells was done by incubating live embryos for 5 min in a 200 μM solution of Daspei (Sigma) in + chorion. After rinsing twice in + chorion, embryos were anesthetized using MS222 and mounted in methylcellulose.

## Supporting Information

Figure S1 Design of Morpholinos Targeting the miR-205 Precursor (224 KB TIF)

Figure S2 Morpholino-Mediated Knockdown of miR-30c (A) Design of morpholinos targeting the miR-30c precursor.
(B) Northern analysis of miR-30c expression in 24-h-old embryos injected with different morpholinos targeting the miR-30c precursor.
(C) Alignment of the precursor of miR-30 family miRNAs.
(784 KB TIF)

Table S1 Morpholinos Used in This Study (21 KB XLS)

Table S2 miRNA Sequences (13 KB XLS)

## Abbreviations

dpf: days postfertilization
GFP: green fluorescent protein
hpf: hours postfertilization
LNA: locked nucleic acid
miRNA: microRNA
MO: morpholino oligonucleotide
RT-PCR: reverse transcriptase PCR
