# Expression of Concern: Targeted Inhibition of miRNA Maturation with Morpholinos Reveals a Role for miR-375 in Pancreatic Islet Development

**DOI:** 10.1371/journal.pbio.3001631

**Published:** 2022-04-29

**Authors:** The PLOS Biology Editors

Following the publication of this article [1], concerns were raised regarding results presented in Figs 1, 2, 5, 6 and S2. Specifically,

- The Fig 1A wild type 5S RNA 24-, 48-, and 72-hour results appear similar to the Fig 3B U6 snRNA skin, liver, and gut results presented in [2] respectively. In addition, the Fig 1A miR-206 MO 5S RNA 24-, 48, and 72-hour results appear similar to the Fig 3B U6 snRNA muscle, gills, and fins results presented in [2] respectively. The corresponding author indicated that the experiments presented in Fig 1A of [1] and the experiments presented in Fig 3B of [2] were carried out simultaneously and that the wrong data have been included in their *PLOS Biology* article. The original data underlying the Fig 1A results are no longer available, but the results presented in Fig 1A have been confirmed by the results presented in Fig 1B. In addition, this experiment was partially repeated at that time, confirming effective knockdown of miR-206 by miR-206 MO in the early embryo. The partial repeat experiment data were recovered and included in the S1 File below. In the updated Fig 1 below, the Fig 1A panel has been removed.

- The following panels display vertical irregularities suggestive of image splicing.
  ○ Fig 2B miR-205 panel between lanes 3–4, and lanes 4–5.
  ○ Fig 2B 5S RNA panel between lanes 3–4, and lanes 4–5.
  ○ Fig 2C miR-205 panel between lanes 4–5, lanes 8–9, lanes 10–11, and lanes 12–13.
  ○ Fig 2C 5S RNA panel between lanes 4–5, lanes 8–9, and lanes 12–13.
  ○ S2B Fig miR-30c panel between lanes 1–2, lanes 2–3, lanes 4–5, and lanes 6–7.
  ○ S2B Fig 5S RNA panel between lanes 1–2, lanes 2–3, lanes 4–5, and lanes 6–7.

**Fig 1:**
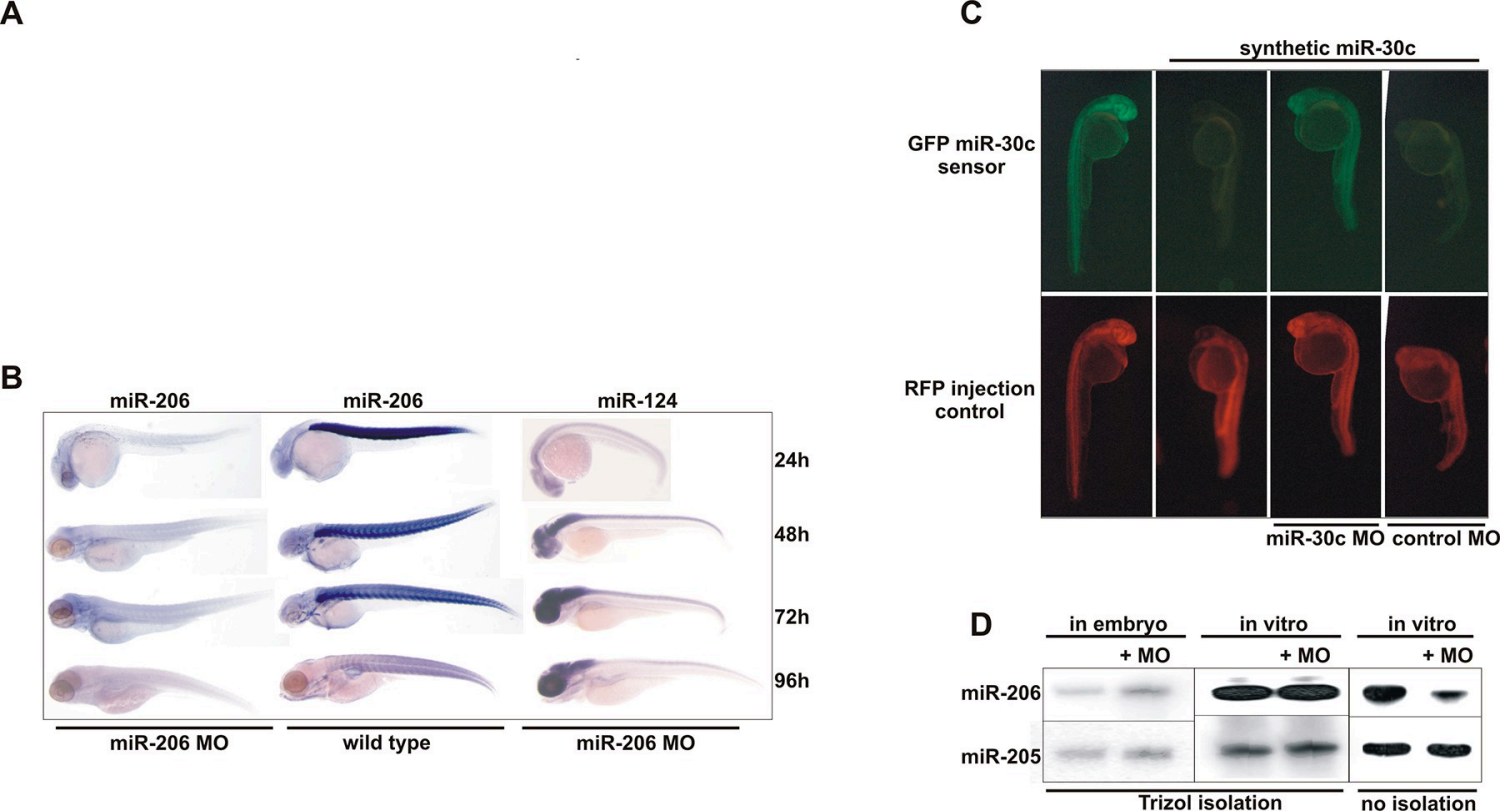
Morpholinos Targeting the Mature miRNA Deplete the Zebrafish Embryo of Specific miRNAs. (A) Removed panel previously showing Northern blot for miR-206 in wild-type and MO miR-206–injected embryos at 24, 48, and 72 hpf. (B) In situ analysis of miR-206 and miR-124 expression in different stage embryos after injection of MO miR-206. (C) Effect of a morpholino targeting miR-30c on a silencing assay with miR-30c and a responsive GFP sensor construct. (D) In vivo and in vitro effects of a morpholino on the stability and RNA extraction of a synthetic miR-206 duplex. miR-205 serves as a loading control.

**Fig 2:**
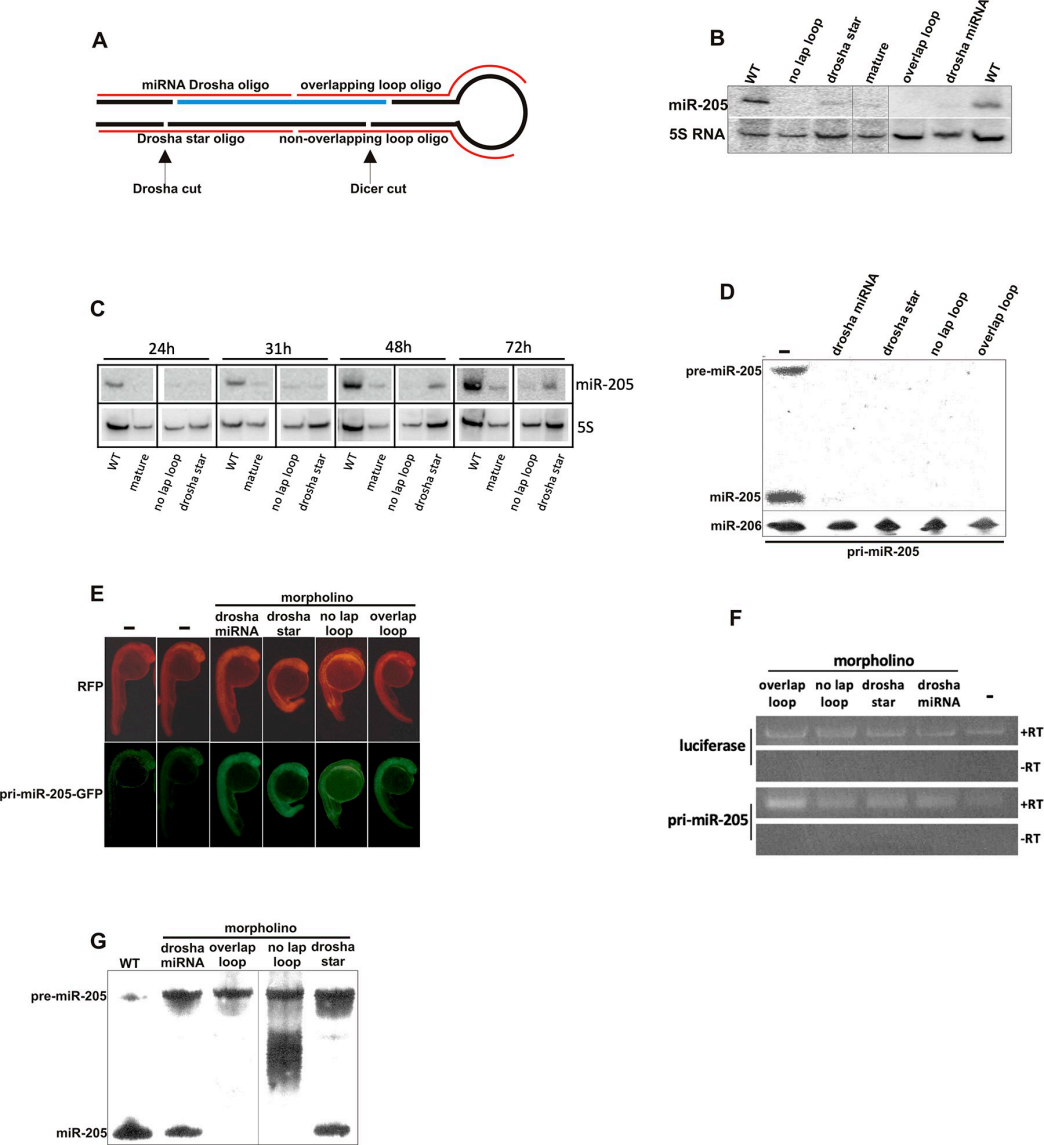
Morpholinos Targeting the Precursor miRNA Interfere with miRNA Maturation. (A) Design of morpholinos targeting the precursor miRNA. (B) Northern blot analysis of miR-205 in 30-h-old embryos injected with different morpholinos against pri-miR-205. 5S RNA serves as a loading control. (C) Time series of miR-205 expression after injection of mature, no lap loop, and drosha star morpholinos against pri-miR-205. (D) Northern blot analysis of miR-205 derived from embryos injected with a GFP-pri-miR-205 transcript and four different morpholinos targeting pri-miR-205. Co-injected miR-206 serves as an injection and loading control. Embryos were collected 8 h after injection. (E) GFP expression in 24-h embryos injected with morpholinos and a GFP-pri-miR-205 construct as used in (C). Pri-miR-205 is positioned just upstream of the polyA signal in the 3′ UTR of the GFP mRNA. Red fluorescent protein (RFP) serves as an injection control. (F) RT-PCR analysis of injected GFP-pri-miR-205 mRNA with (+) and without (−) co-injected morpholinos. Luciferase serves as an injection control. Embryos were collected 8 h after injection. (G) Northern analysis of the effect of morpholinos on an injected miR-205 precursor. Embryos were collected 8 h after injection. WT, wild type.

**Fig 5:**
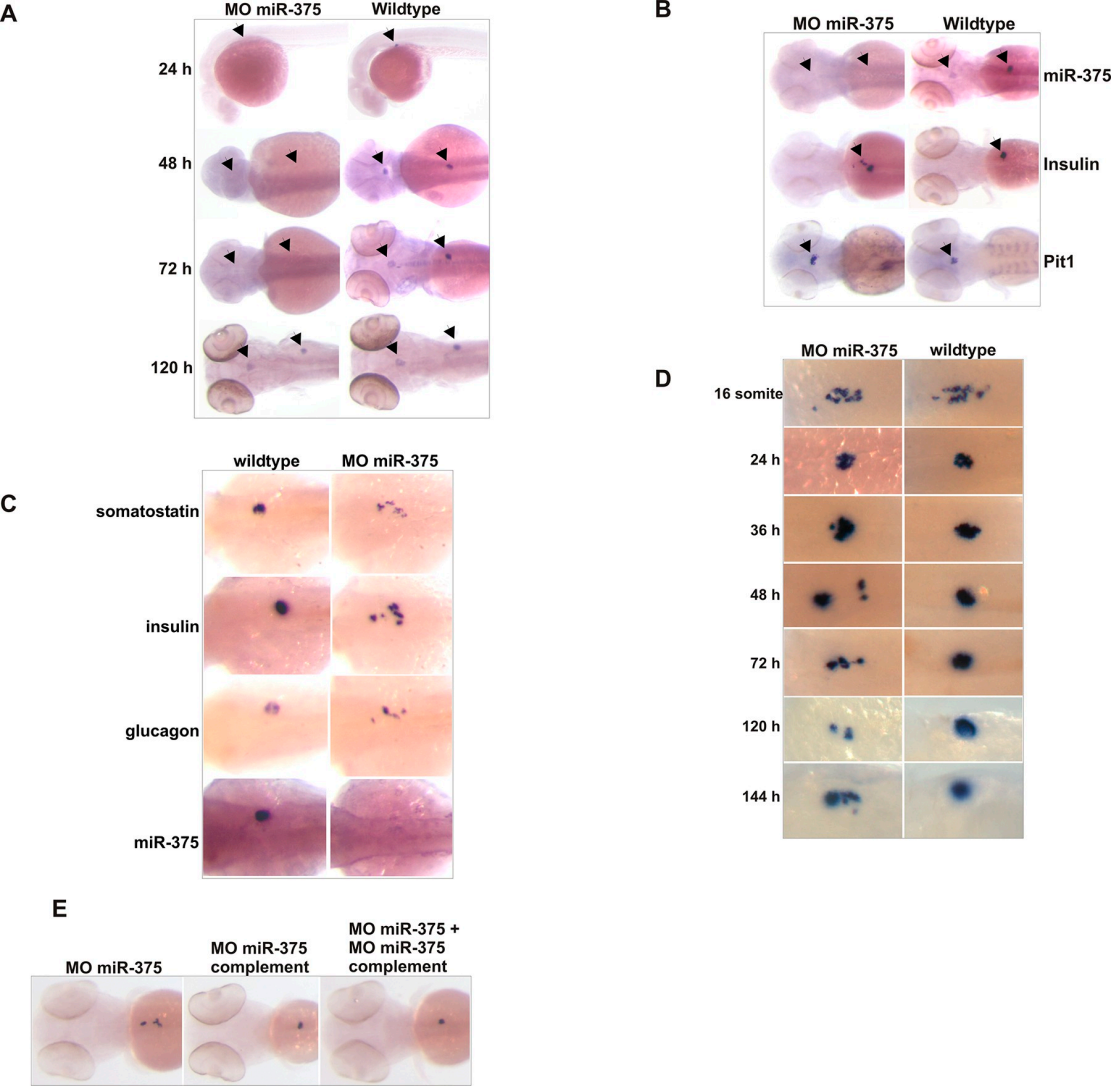
Knockdown of miR-375 Results in Aberrant Migration of Pancreatic Islet Cells. (A) In situ analysis of miR-375 knockdown in MO miR-375–injected embryos and noninjected controls at 24, 48, 72, and 120 hpf. Arrowheads indicate the pituitary gland and the pancreatic islet. (B) In situ analysis of the pancreatic islet (insulin staining) and the pituitary gland (pit1 staining) in miR-375 morphants and noninjected controls. Arrowheads indicate the pituitary gland and the pancreatic islet. (C) In situ analysis of pancreatic islet development in wild-type and morphant embryos using insulin, somatostatin, and glucagon as markers. (D) Time series of insulin expression in wild-type and morphant embryos injected with MO miR-375. The WT 72h panel of Fig 5D is identical to the WT insulin panel of Fig 6A, due to these panels representing the same experimental condition. (E) Insulin expression in 72-hpf embryos injected with MO miR-375 and a complementary morpholino.

**Fig 6:**
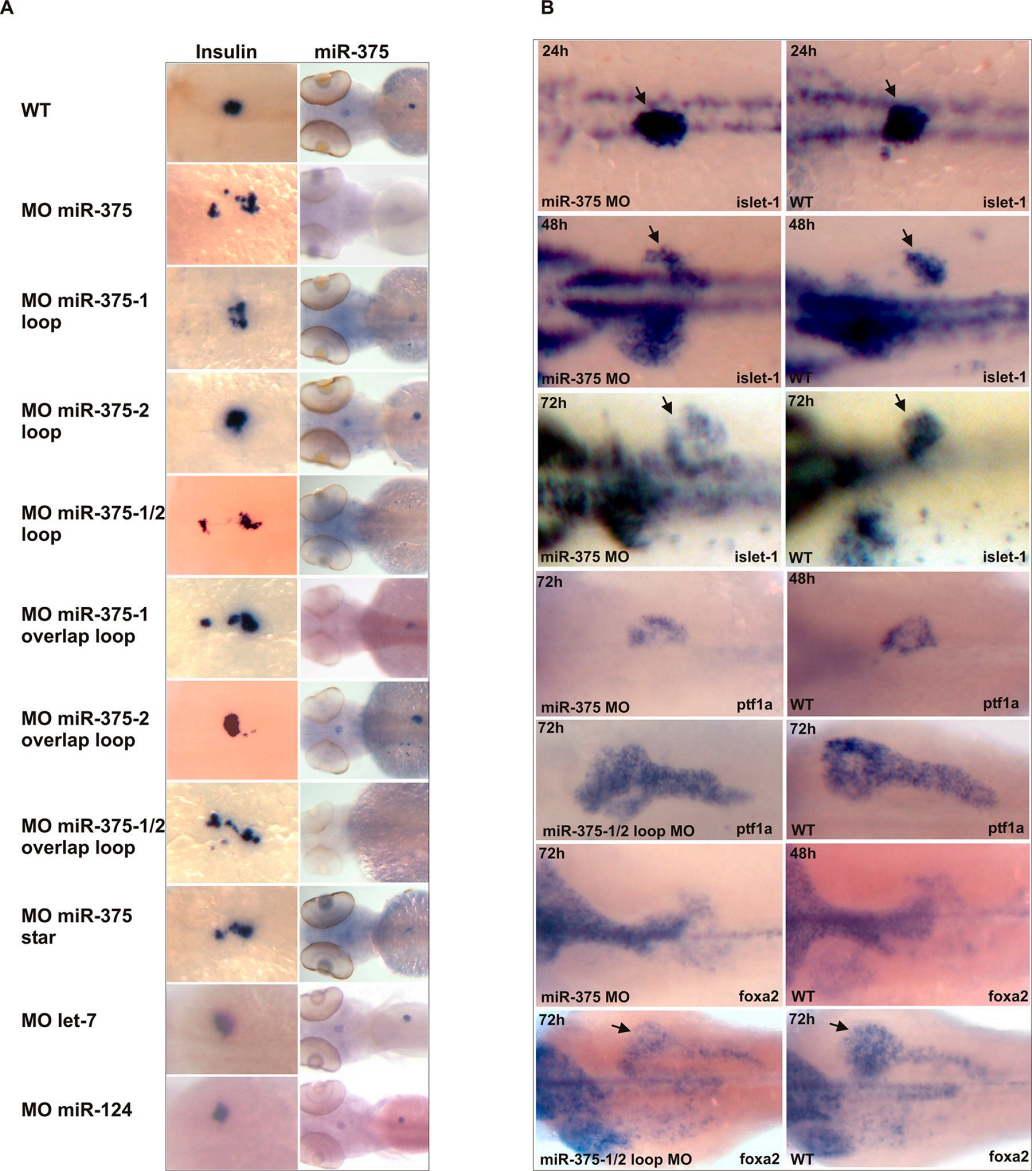
Specific Effects of miR-375 Knockdown on the Development of the Endocrine Pancreas. (A) In situ analysis of miR-375 and insulin expression in 72-hpf embryos injected with morpholinos against the miR-375 precursor and negative control morpholinos for let-7 and miR-124. The WT insulin panel of Fig 6A is identical to the WT 72h panel of Fig 5D, due to these panels representing the same experimental condition. (B) Expression of islet1, foxa2, and ptf1a in wild-type and miR-375 knockdown embryos. Arrows indicate the pancreatic islet. WT, wild type.

The corresponding author clarified that lanes 1–4 and lanes 5–7 of the miR-205 and 5S RNA panels of Fig 2B originate from separate northern blots, and that a wild-type sample was loaded onto both blots to allow for comparison between the samples. The available underlying data for Fig 2B are provided in the S1 File below, and the journal considers the concerns with this figure resolved. Similarly, the corresponding author clarified that the experiments for the different time points presented in Fig 2C were obtained from separate blots with a wild-type sample loaded on each blot to allow for comparison between the blots. During their reassessment of this Figure, the corresponding author noted there were errors in the 5S RNA results presented in Fig 2C. In the updated Fig 2 below, the correct 5S RNA results have been presented and the panels 2B and 2C have been corrected to clarify that the panels have been prepared using spliced data obtained from separate blots. The available data underlying Fig 2C are provided in the S1 File below.

- In Fig 2D there appears to be a horizontal irregularity suggestive of image splicing between the miR-205 results and the miR-206 results. The corresponding author noted that miR-205 and miR-206 were run on separate blots due to their similarity in molecular weight. In the updated Fig 2 below, the 2D panel has been corrected to clarify that the panel has been prepared using data originating from separate blots. The original data underlying Fig 2D are provided in the S1 File below, and the journal considers the concerns with this figure resolved.
- The luciferase -RT panel of Fig 2F appears similar to the pri-miR-205 -RT panel of Fig 2F when rotated 180°. The corresponding author clarified that the luciferase and pri-miR-205 samples were mixed and loaded into the same lanes to allow for a direct comparison within the lanes. The authors noted that the -RT panels originate from the same blot, but that an error was made during the preparation of the figure so that the orientation of one of the negative control panels is incorrect. The authors provided the original uncropped blot underlying the results presented in this panel, which supports the overall results and conclusions presented in this figure. In the updated Fig 2 below, the 2F panel has been corrected to represent the correct -RT results. The original data underlying Fig 2F are provided in the S1 File below.
- The WT 72h panel of Fig 5D is identical to the WT insulin panel of Fig 6A. The corresponding author confirms that these panels were duplicated because they represent the same experimental conditions. The Figure legends for Fig 5D and Fig 6A have been updated to clarify the duplication, and the journal considers the concerns with this figure resolved.

- In S2B Fig there appear to be vertical irregularities suggestive of image splicing between lanes 1–2, lanes 2–3, lanes 4–5, and lanes 6–7. The corresponding author noted that these results were obtained from two separate blots that were run in parallel. In the updated S2 Fig in the Supporting Information, the panel has been corrected to clarify that the panel has been prepared using spliced data obtained from separate blots. The original blots underlying the S2 Fig results are provided in the S1 File below, and the journal considers the concerns with this figure resolved.

The *PLOS Biology* Editors issue this Expression of Concern in light of the concerns raised with Fig 1A, which cannot be corrected in the absence of underlying data, as well as due to the accumulation of image reporting errors and aesthetic image issues involving Figs 2 and S2.

## Supporting information

S1 File Available underlying data. (PDF)

S2 Fig Morpholino-Mediated Knockdown of miR-30c (A) Design of morpholinos targeting the miR-30c precursor. (B) Northern analysis of miR-30c expression in 24-h-old embryos injected with different morpholinos targeting the miR-30c precursor. (C) Alignment of the precursor of miR-30 family miRNAs.
(TIF)
