# Supplementary material for: Expression of Concern: Targeted Inhibition of miRNA Maturation with Morpholinos Reveals a Role for miR-375 in Pancreatic Islet Development
Source: PLoS Biol. 2022 Apr 29;20(4):e3001631. doi: 10.1371/journal.pbio.3001631 (PMC9053783; doi:10.1371/journal.pbio.3001631)

Partial repeat data Figure 1A

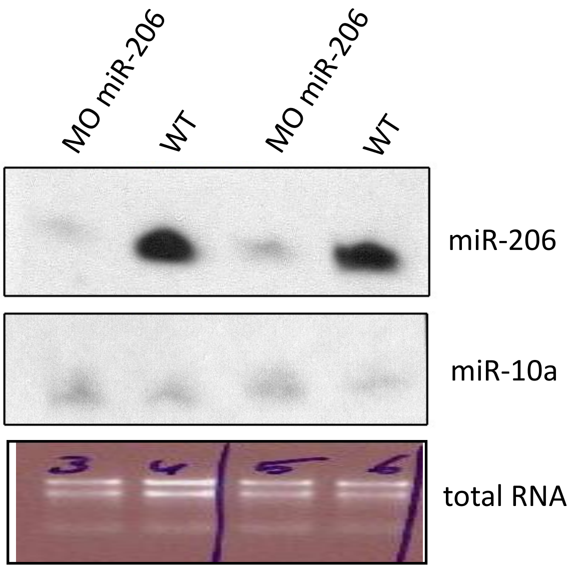

This figure represent partial repeat data for knockdown of miR-206 using a MO miR-206. The exact embryo development timepoint could not be identified for each of the lanes, but is either 24hpf, 48hpf or 72hpf. The data show that there is a strong effect of a MO miR-206 on expression of miR-206 in the zebrafish embryo, similarly to what is shown in the original Figure 1A in the published paper.

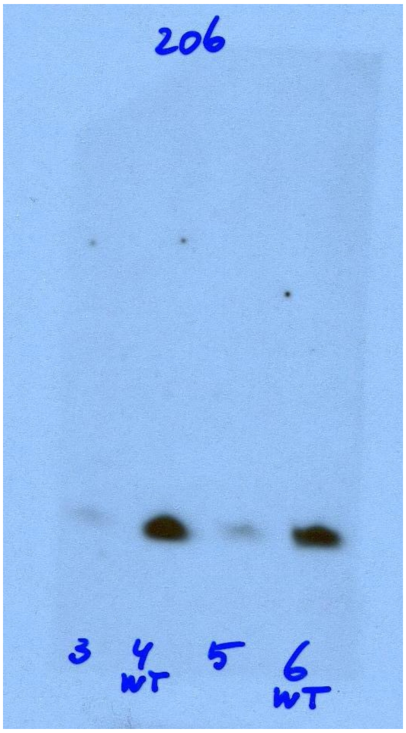

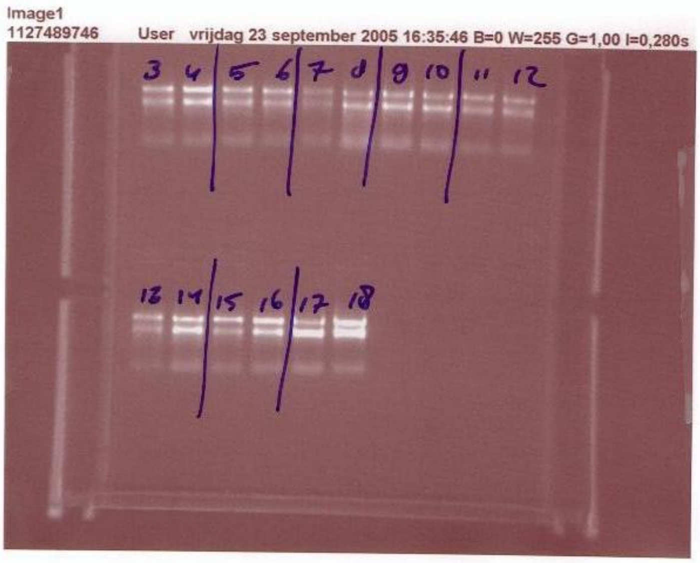

Partial original data Figure 1B

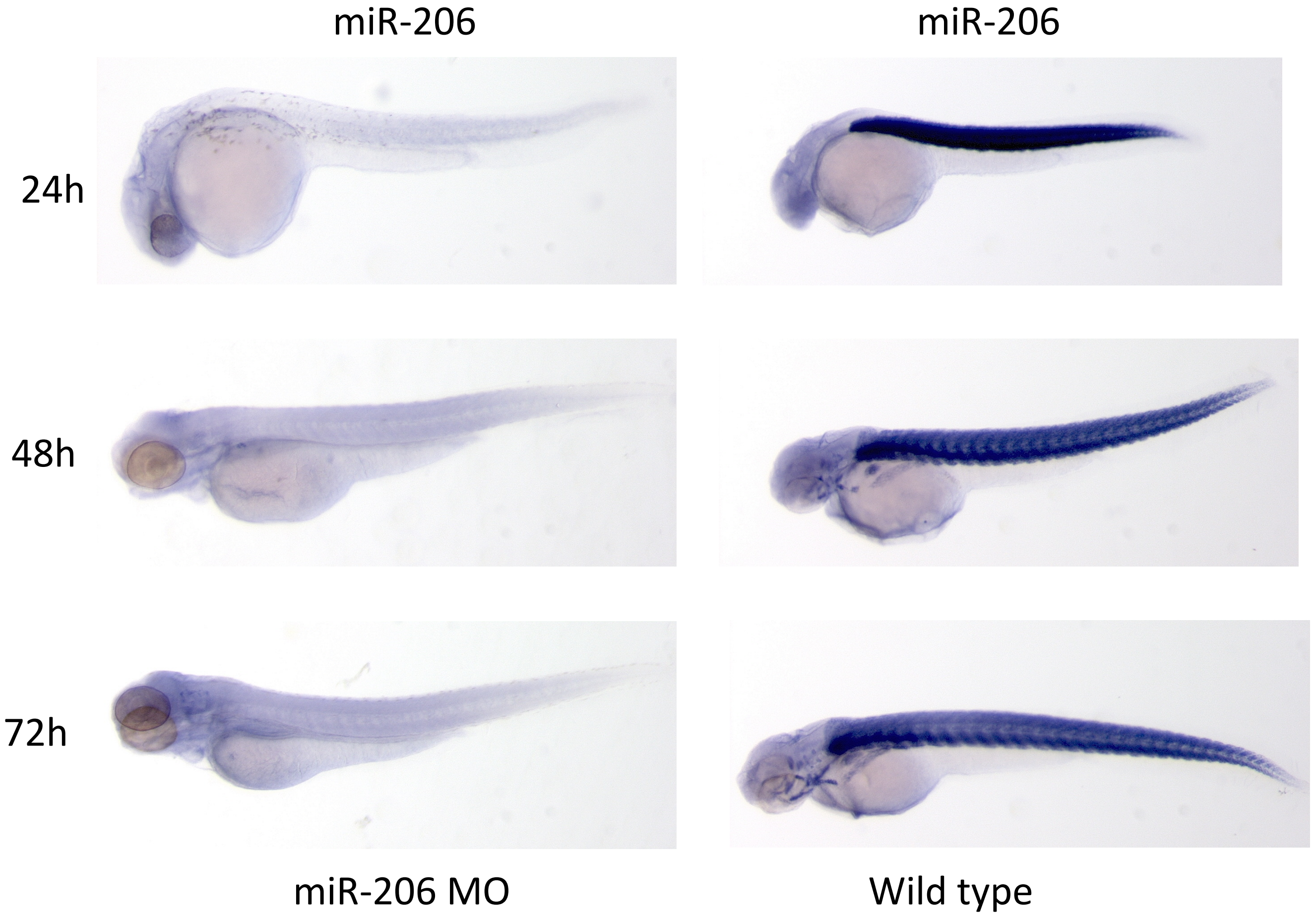

Original data Figure 1C

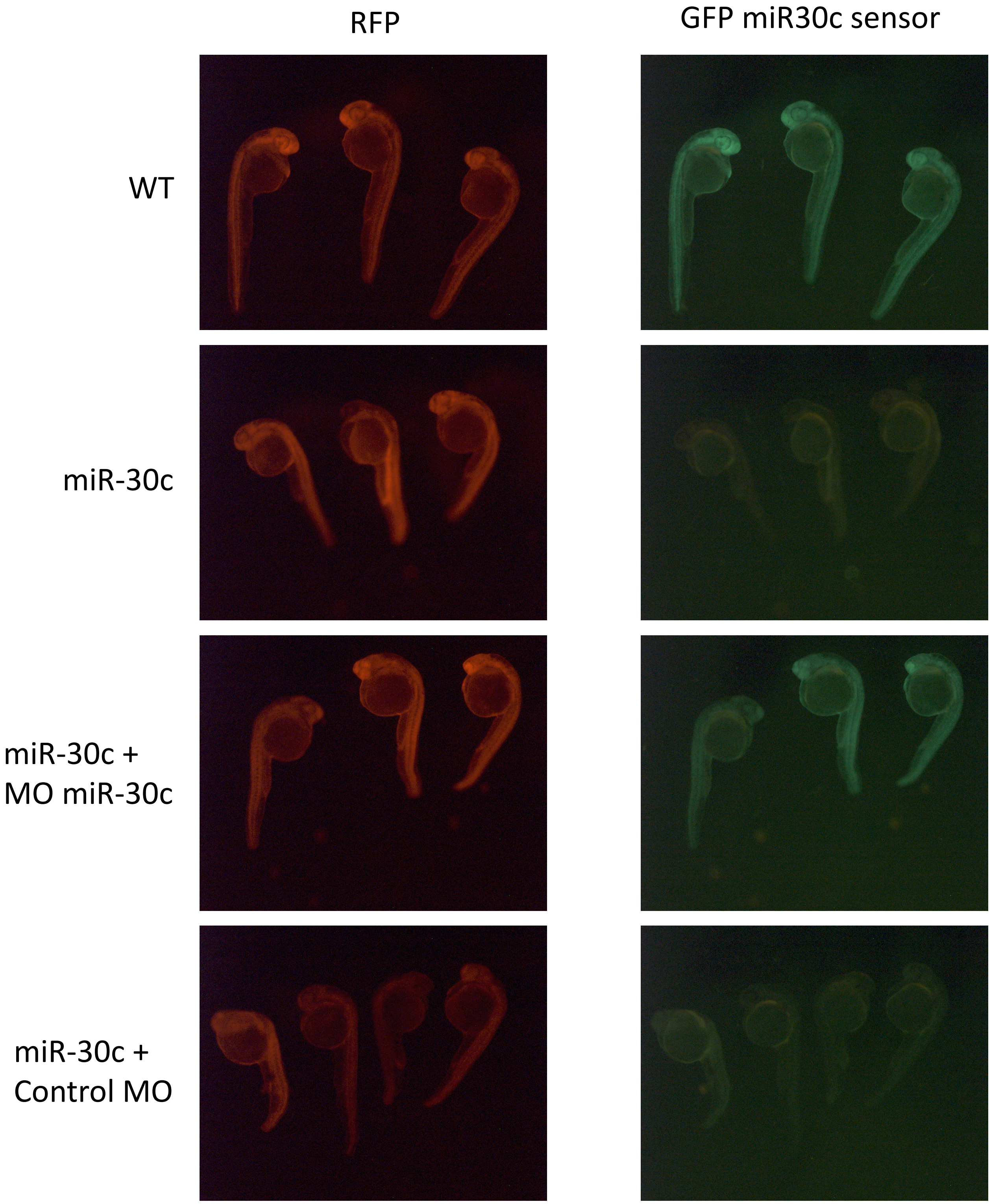

Of note: only one of the three imaged fishes per condition has been used for the publication.

Partial original data Figure 1D

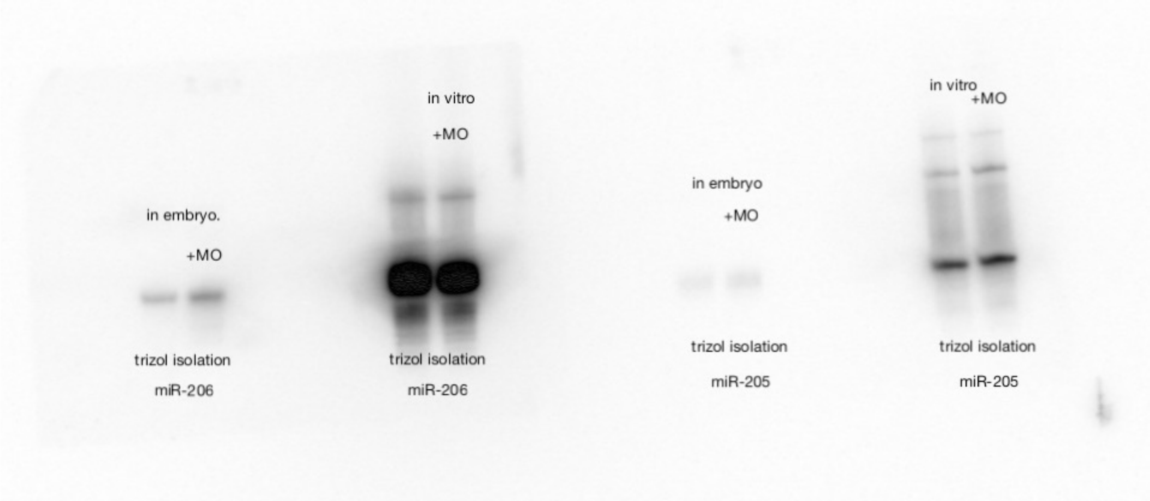

Partial original data Figure 1D with annotation. Data for condition ‘no isolation’ could not be found anymore. These data indicate that the miR-205 and miR-206 expression was analyzed on separate blots.

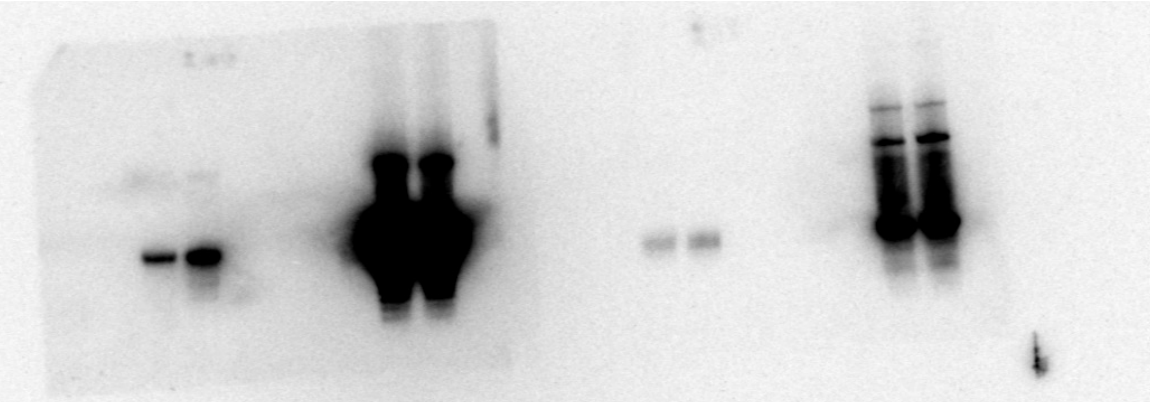

Partial original data Figure 1D based on overexposure of a signal to a film. The blot is the same as above, except from a longer exposure.

Partial original data Figure 2B

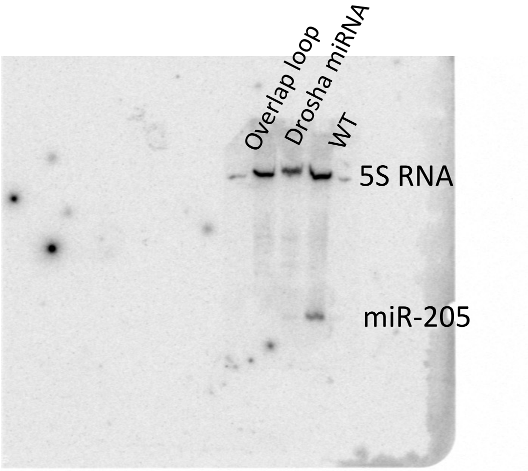

Partial original data for Figure 2B (right part).

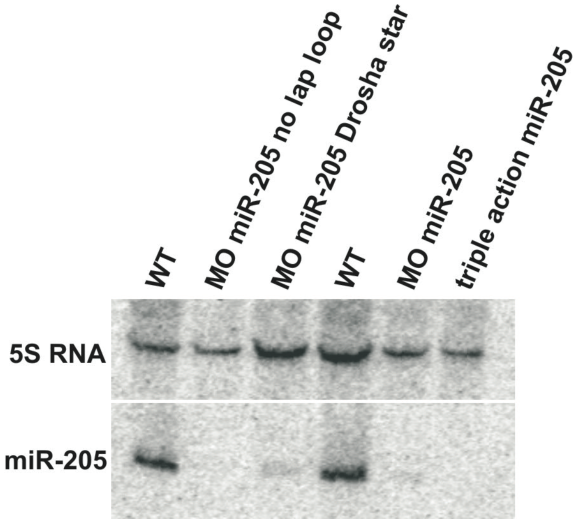

Partial data Figure 2B (left part).

Original data Figure 2C
Correctly processed data for Figure 2C

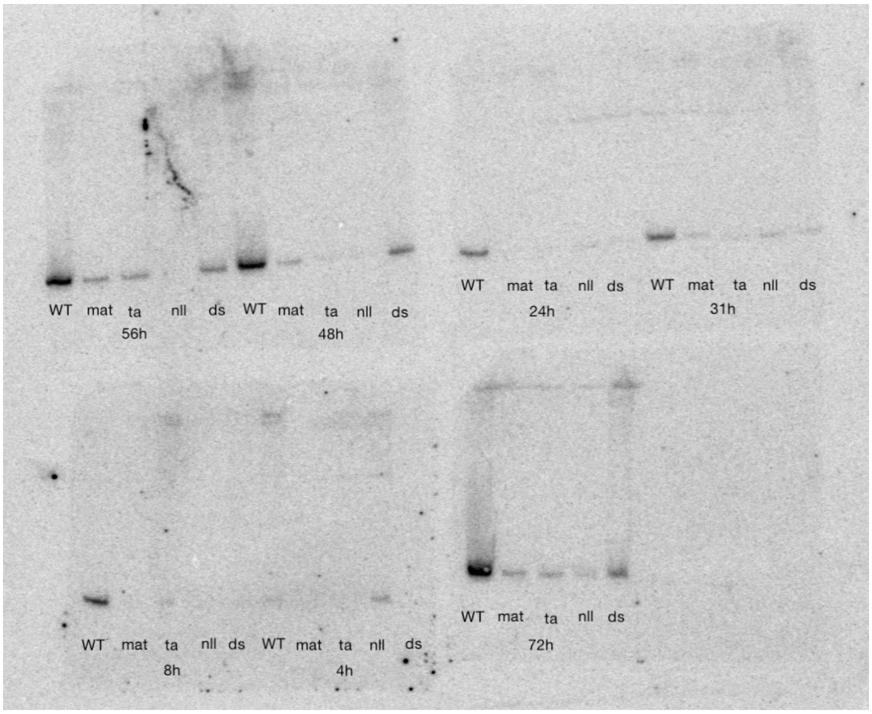

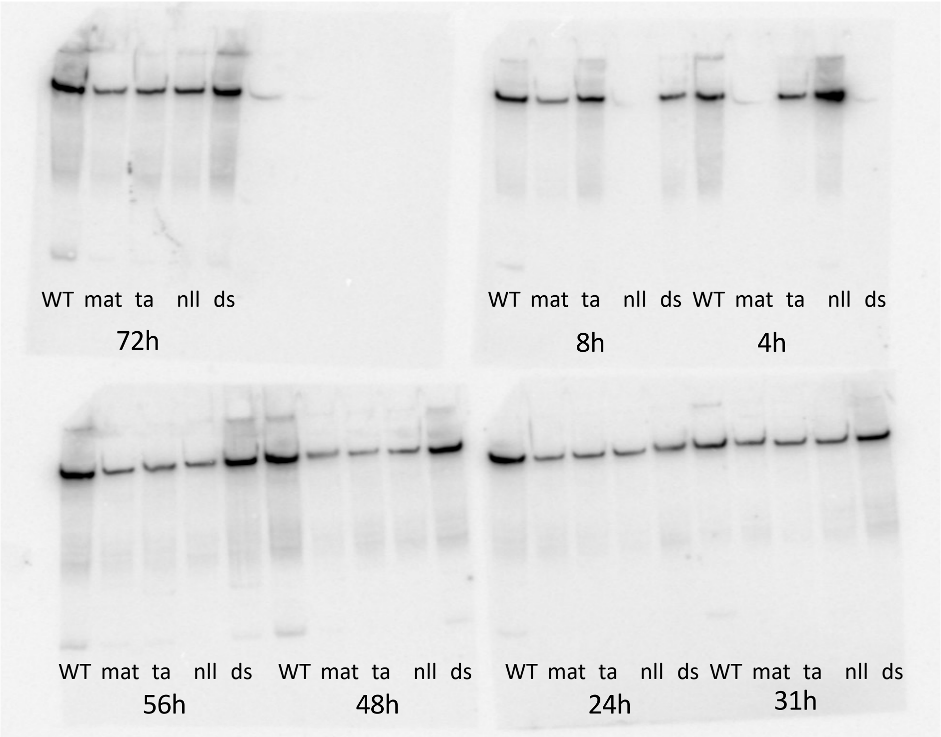

Corrected Figure 2C

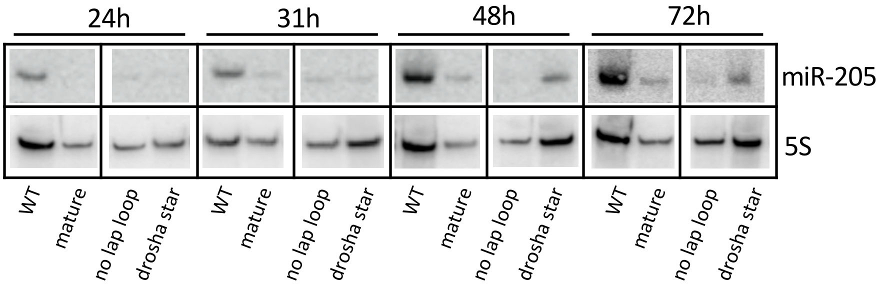

Original data Figure 2D

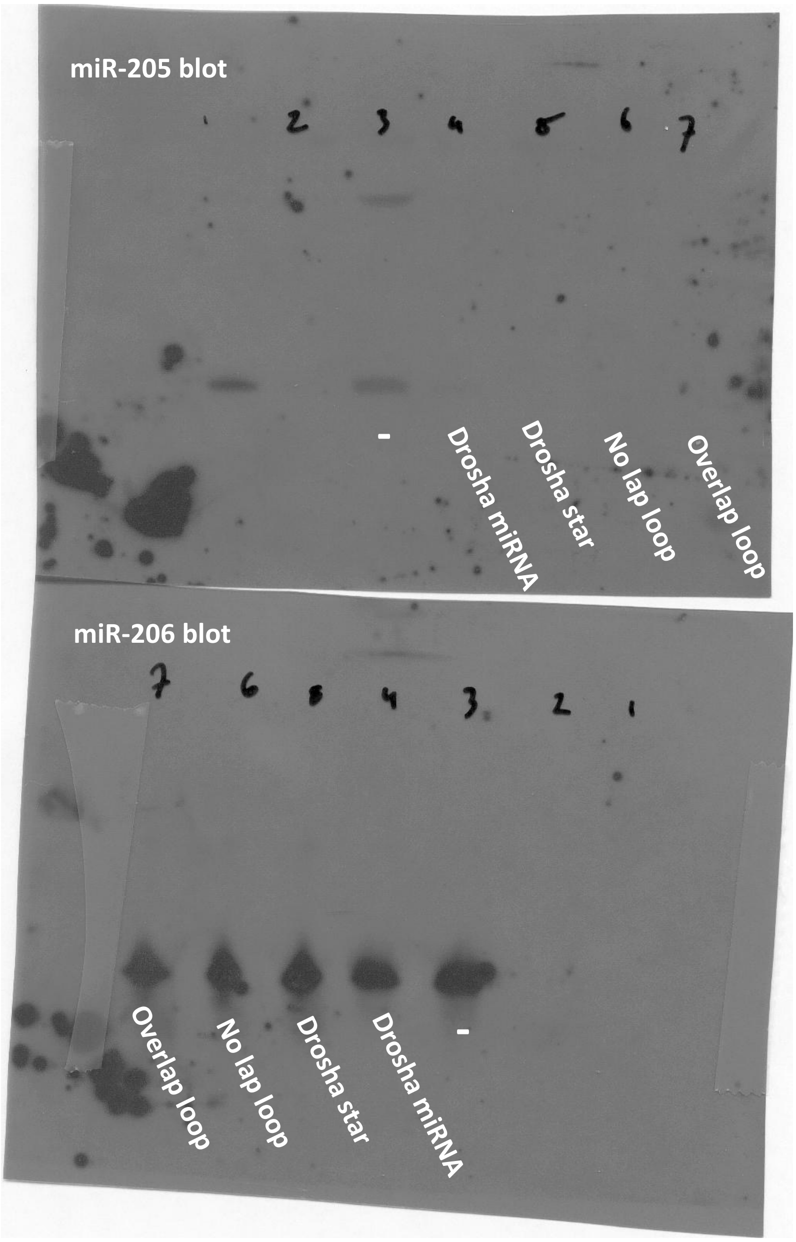

Original data Figure 2F

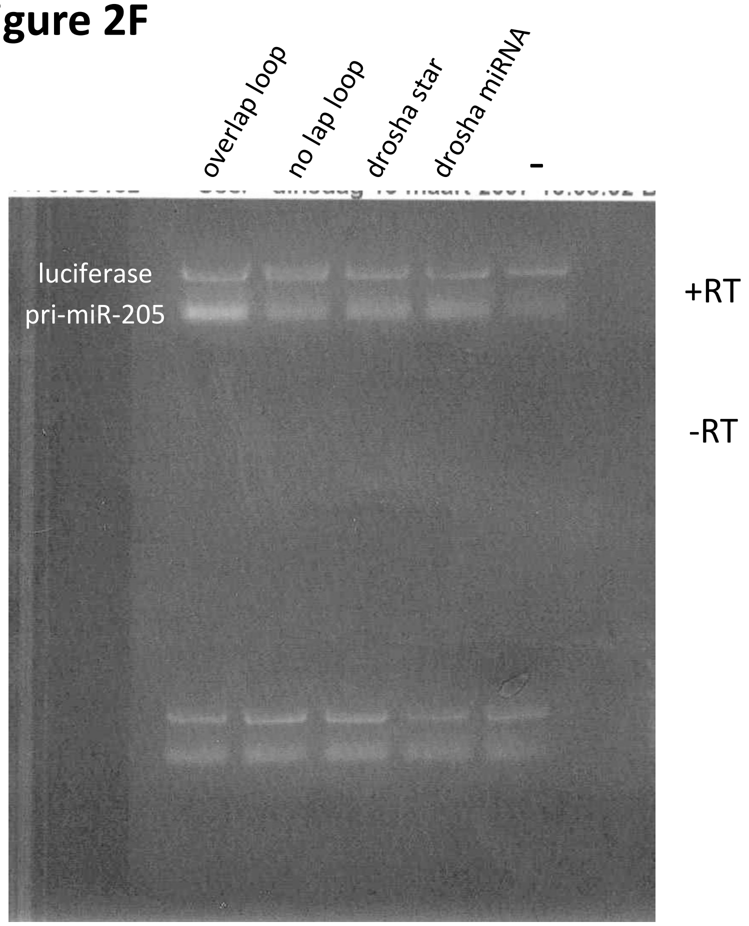

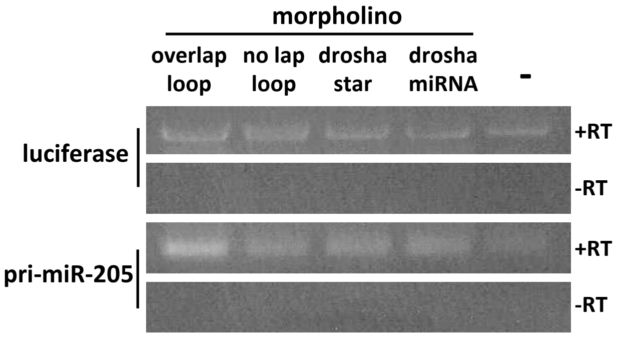

Corrected Figure 2F

Original data Figure 2G

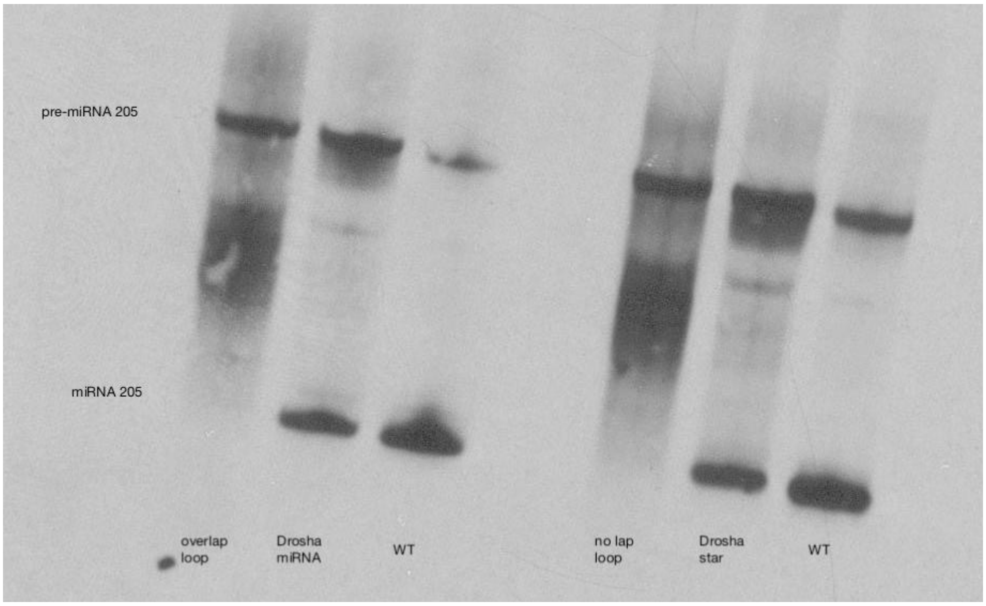

Original data Figure 5C

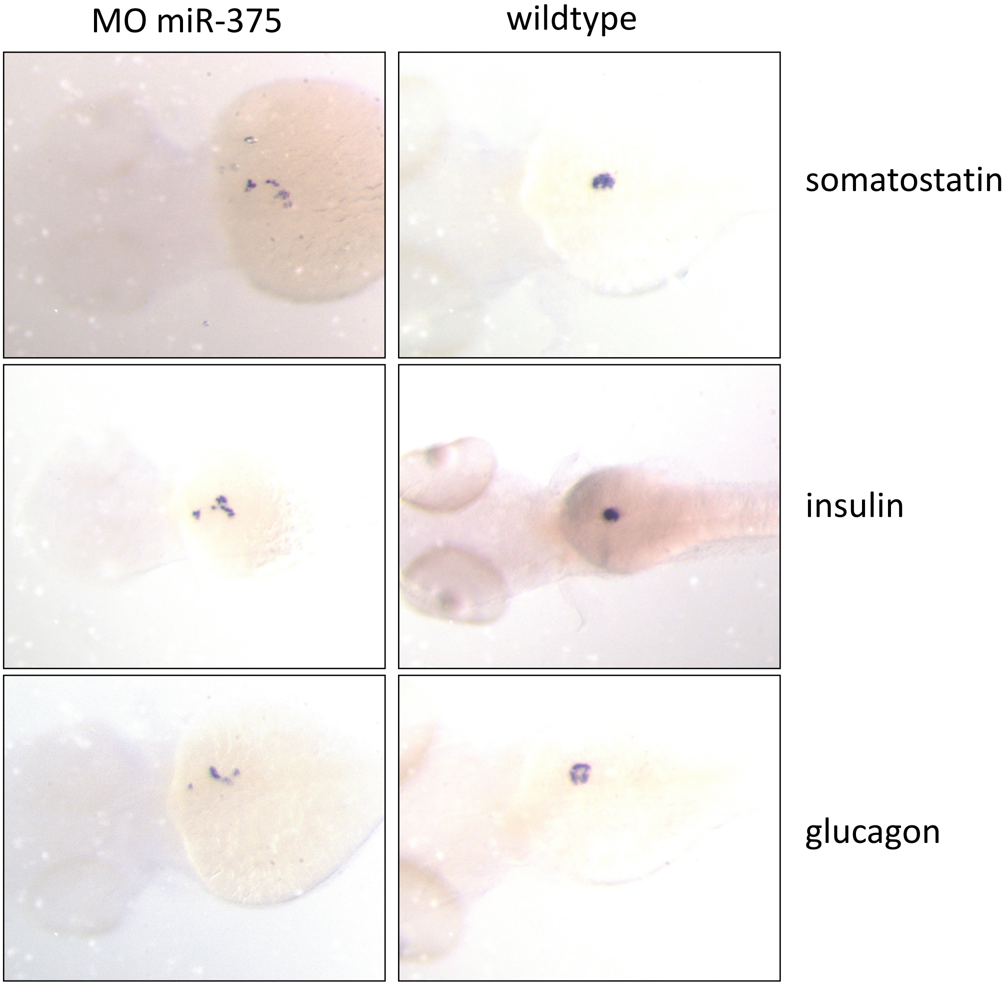

Original data Figure 5D

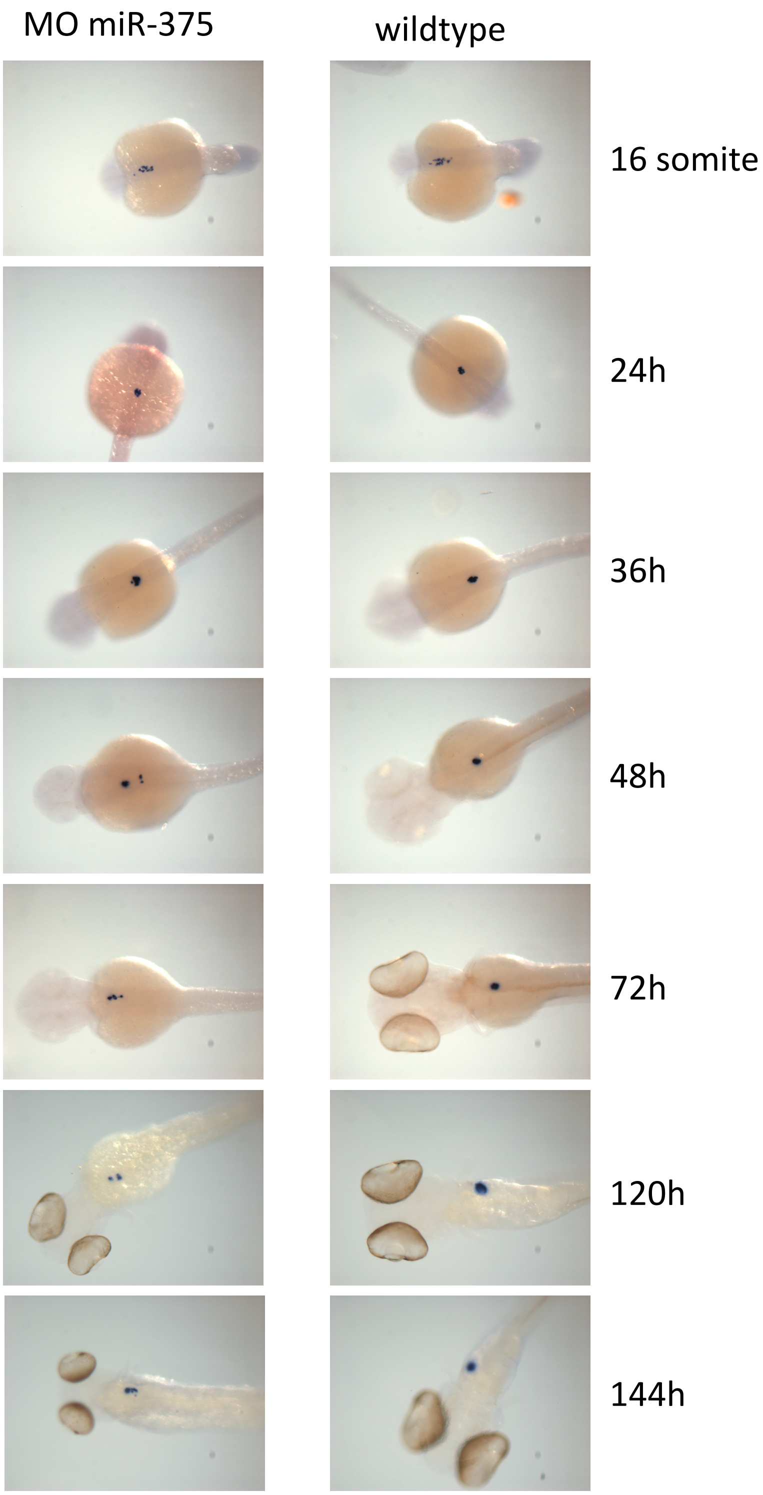

Original data Figure 5E

MO miR-375

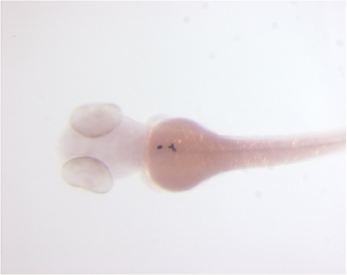

MO miR-375
complement

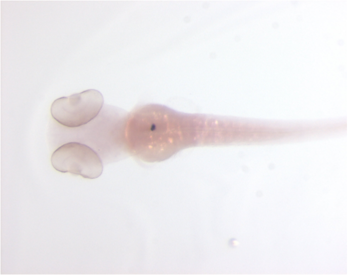

MO miR-375
complement

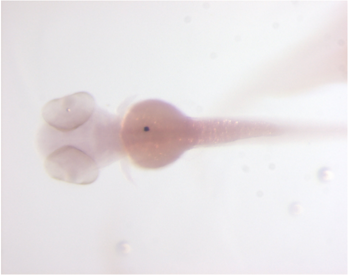

Partial original data Figure 6A

Insulin | miR-375

MO miR-375-1 loop

MO miR-375-2 loop

MO miR-375-1/2 loop

MO miR-375-1 overlap loop

MO miR-375-2 overlap loop

MO miR-375-1/2 overlap loop

MO miR-375 star

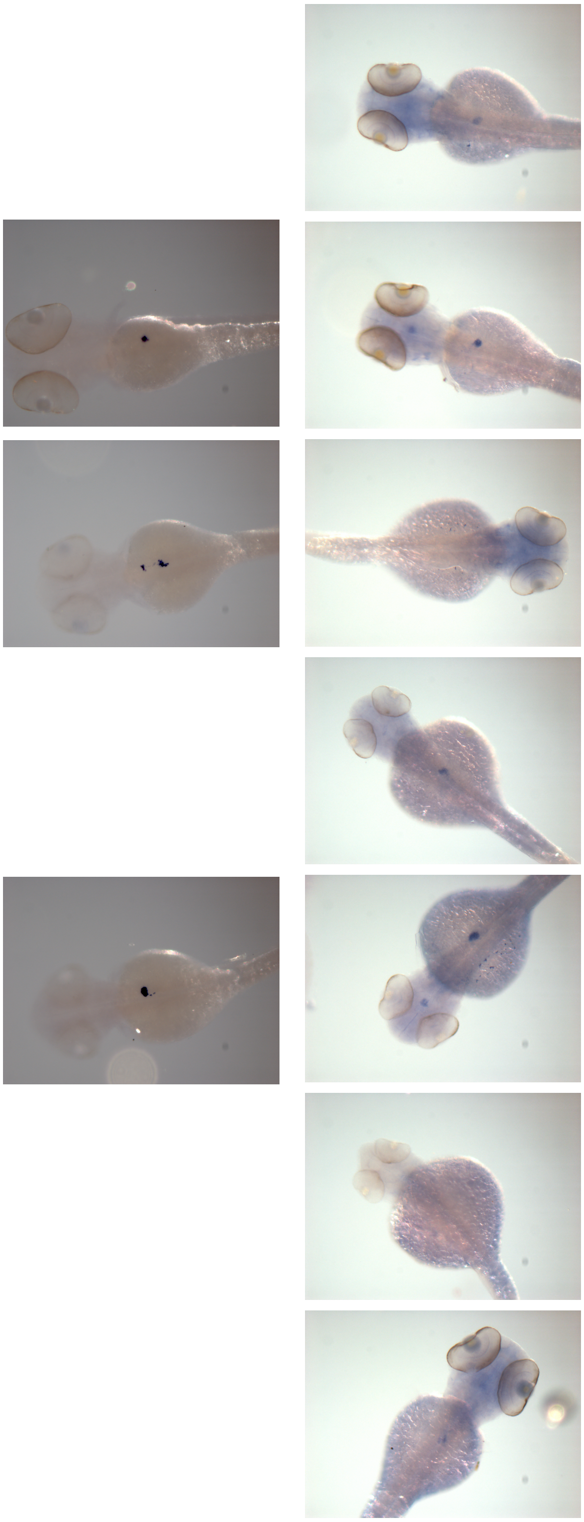

Original data Figure 6B

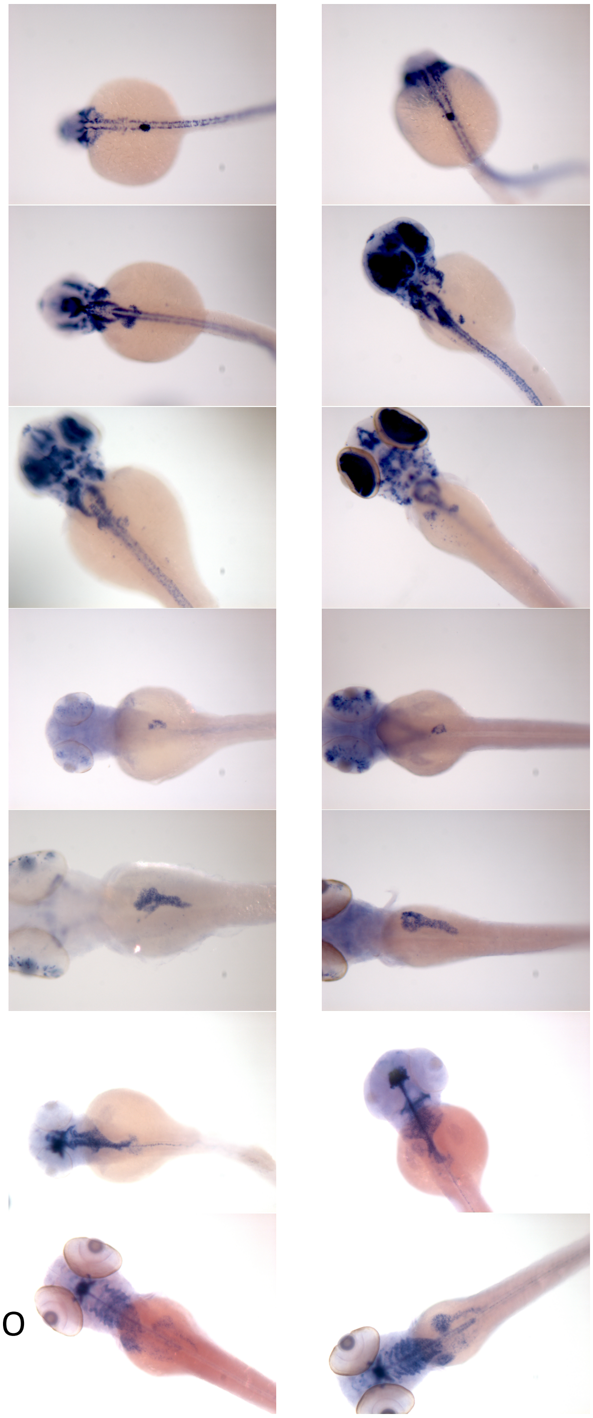

24h
miR-375 MO
islet-1

24h
WT
islet-1

48h
miR-375 MO
islet-1

48h
WT
islet-1

72h
miR-375 MO
islet-1

72h
WT
islet-1

72h
miR-375 MO
ptf1a

48h
WT
ptf1a

72h
miR-375-1/2 MO
ptf1a

72h
WT
ptf1a

72h
miR-375 MO
foxa2

48h
WT
foxa2

72h
miR-375-1/2 loop MO
foxa2

72h
WT
foxa2

Complete original data Figure S2B

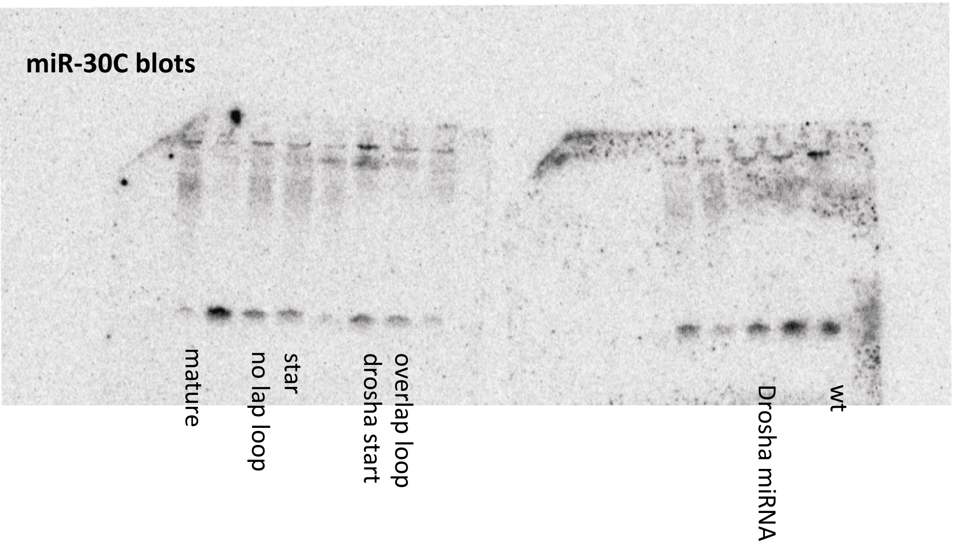

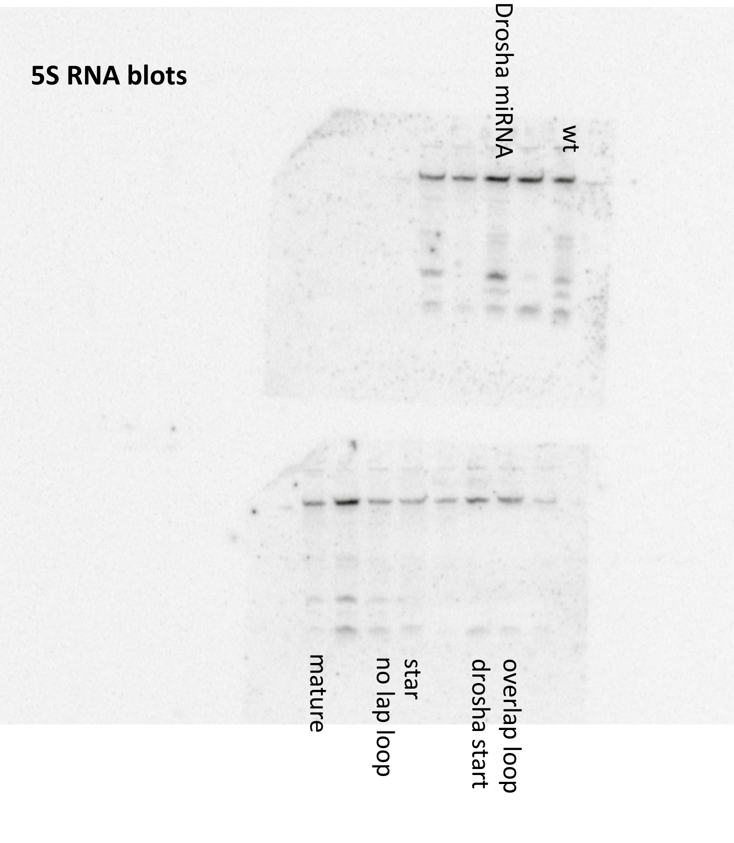

Supplement: S1 File — (PDF) [file pbio.3001631.s001.pdf]
