# Supplementary figures and images for: Expression of Concern: Targeted Inhibition of miRNA Maturation with Morpholinos Reveals a Role for miR-375 in Pancreatic Islet Development
Source: PLoS Biol. 2022 Apr 29;20(4):e3001631. doi: 10.1371/journal.pbio.3001631 (PMC9053783; doi:10.1371/journal.pbio.3001631)

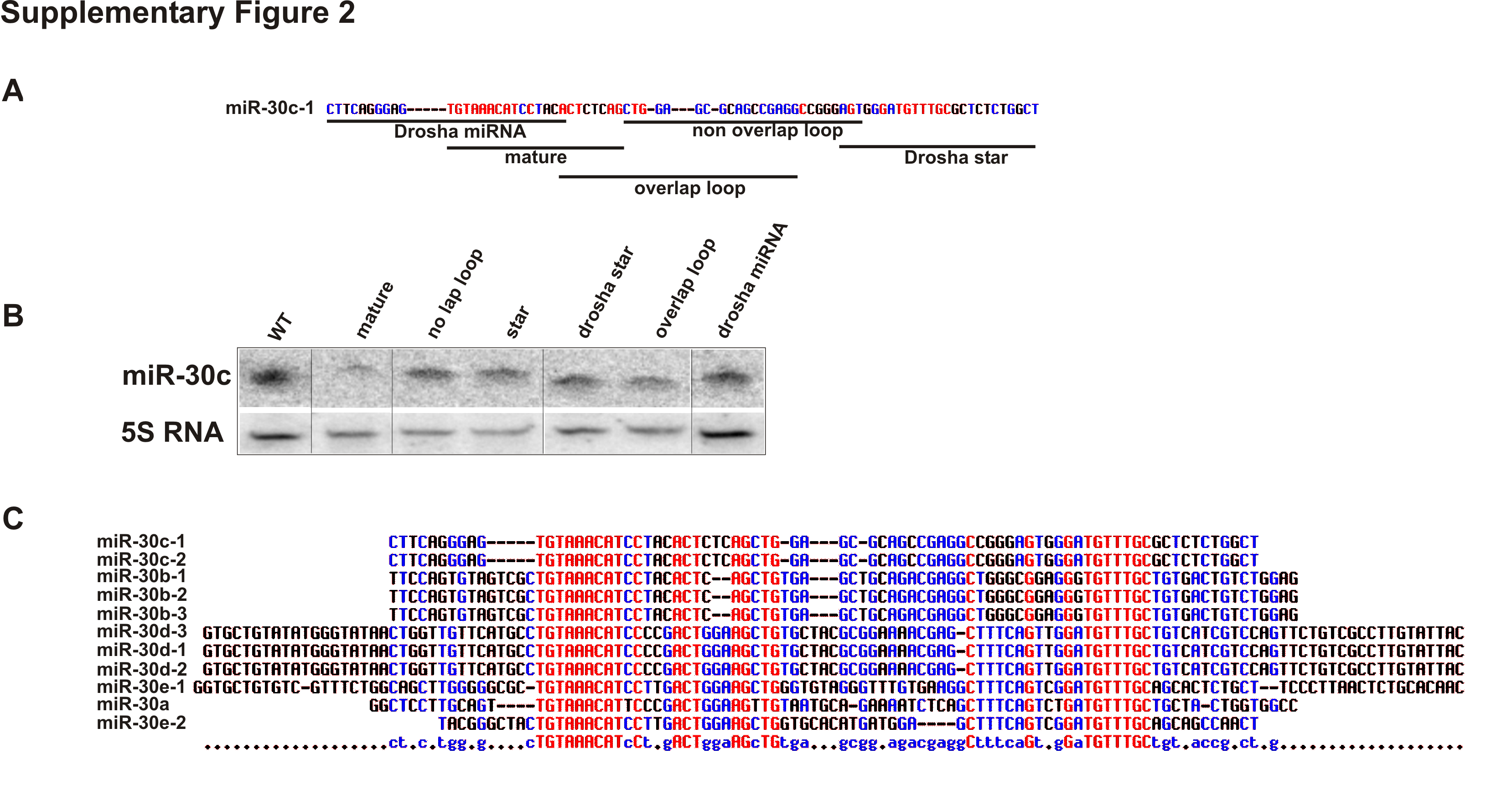

Supplement: S2 Fig — Morpholino-Mediated Knockdown of miR-30c (A) Design of morpholinos targeting the miR-30c precursor. (B) Northern analysis of miR-30c expression in 24-h-old embryos injected with different morpholinos targeting the miR-30c precursor. (C) Alignment of the precursor of miR-30 family miRNAs. (TIF) [file pbio.3001631.s002.tif]
